# Supplementary material for: Consensus model of a cyanobacterial light-dependent protochlorophyllide oxidoreductase in its pigment-free apo-form and photoactive ternary complex
Source: Commun Biol. 2019 Sep 25;2:351. doi: 10.1038/s42003-019-0590-4 (PMC6761149; doi:10.1038/s42003-019-0590-4)
Supplement: Supplementary file 1 — Supplementary Information [file 42003_2019_590_MOESM1_ESM.pdf]

Supplementary Figures

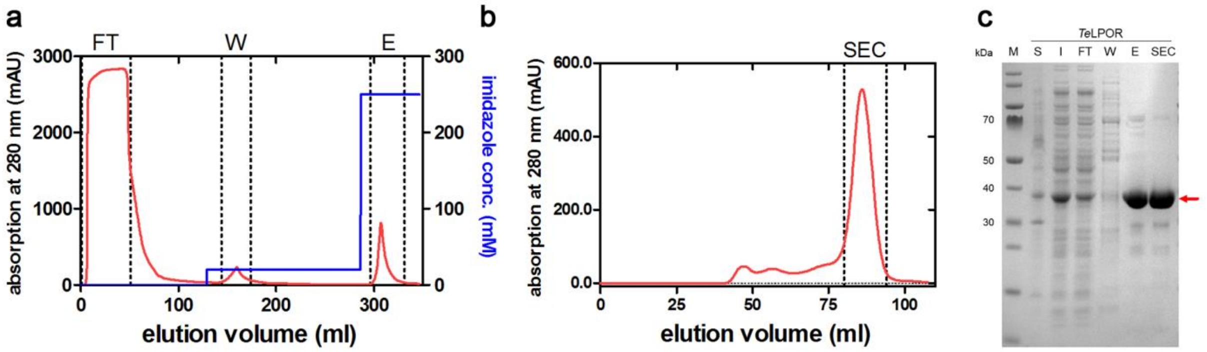

Supplementary Figure 1: Exemplary purification of the *Te*LPOR apoprotein by **a,** IMAC, **b,** SEC and **c,** SDS-PAGE analysis of the purification progress. Protein fractions that were collected during the purification procedure and later analyzed by SDS-PAGE (10-12% pre-cast Bis-Tris gel; MES running buffer) are marked by dashed lines. FT: flow-through fraction, W: wash fraction and elution fraction of IMAC purification step; SEC: elution fraction of SEC run. The fractions collected during the SEC run were pooled and used as sample for the preparation of the corresponding apo- and holoprotein SAXS samples. The blue line in a shows the imidazole concentration used during IMAC purification. In c, additionally the soluble protein fraction (S) and the insoluble protein fraction (I) obtained by centrifugation after cell lysis are shown. The expected theoretical molecular mass computed from the amino acid sequence (38,014 Da) is marked by a red arrow in panel C. M: PageRuler^{TM} prestained protein ladder, 10-180 kDa (Thermo Fisher Scientific, Waltham, MA, USA)

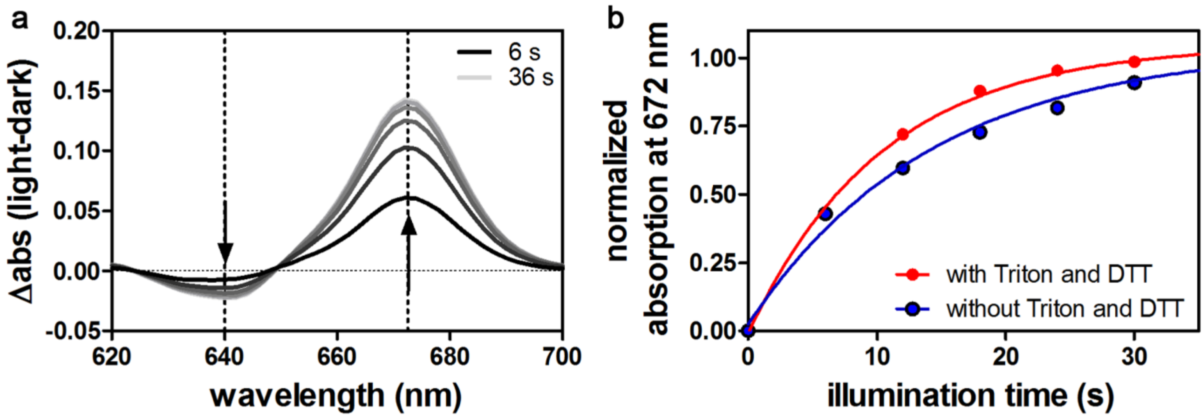

Supplementary Figure 2: a, Light-dark difference spectra illustrating the light-dependent turnover of Pchlide by *Te*LPOR holoprotein samples in the presence DTT and Triton X-100. The *Te*LPOR holoprotein was diluted in reaction buffer and the sample was supplemented with 70 µM DTT and 0.03 % (v/v) Triton X-100. After incubation, the sample was illuminated with blue light at 6 second intervals (6 to 36 seconds; shown in shades of grey). **b,** Comparison of the relative light-dependent Pchlide turnover in the presence (red) and absence (blue) of DTT and Triton. Turnover is illustrated by plotting the normalized increase in absorption at 672 nm, which corresponds to the absorption band of the Chlide product, against the illumination time.

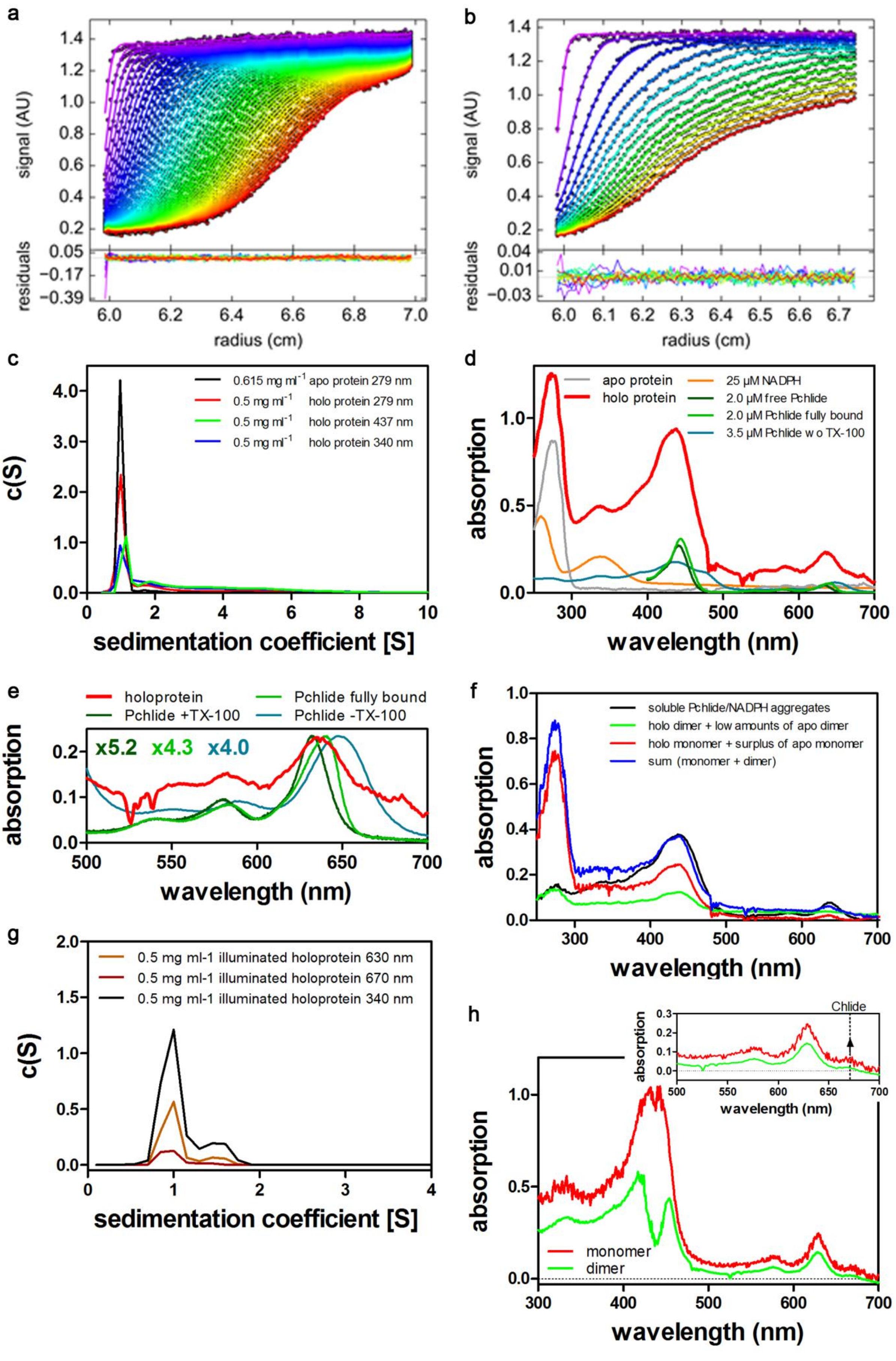
a
signal (AU)
residuals
radius (cm)
b
signal (AU)
residuals
radius (cm)
c
c(S)
0.615 mg ml^{-1} apo protein 279 nm
0.5 mg ml^{-1} holo protein 279 nm
0.5 mg ml^{-1} holo protein 437 nm
0.5 mg ml^{-1} holo protein 340 nm
sedimentation coefficient [S]
d
apo protein
holo protein
25 µM NADPH
2.0 µM free Pchlide
2.0 µM Pchlide fully bound
3.5 µM Pchlide w o TX-100
absorption
wavelength (nm)
e
holoprotein
Pchlide +TX-100
Pchlide fully bound
Pchlide -TX-100
x5.2
x4.3
x4.0
absorption
wavelength (nm)
f
soluble Pchlide/NADPH aggregates
holo dimer + low amounts of apo dimer
holo monomer + surplus of apo monomer
sum (monomer + dimer)
absorption
wavelength (nm)
g
0.5 mg ml-1 illuminated holoprotein 630 nm
0.5 mg ml-1 illuminated holoprotein 670 nm
0.5 mg ml-1 illuminated holoprotein 340 nm
c(S)
sedimentation coefficient [S]
h
Chlide
absorption
wavelength (nm)
monomer
dimer
absorption
wavelength (nm)

Supplementary Figure 3: Multiwavelength absorbance analytical ultracentrifugation (MWA-AUC) analyses. Exemplary sedimentation velocity analysis of **a,** 0.615 mg/mL apoprotein (A) and **b,** 1 mg/mL holoprotein at **a,** 279 nm and **b,** 440 nm (B) with the standard c(s) model. The top panels show raw data (circles) and best fits (lines). For clarity, only every second scan of the data set is shown. The bottom panels display best fit residuals of the plotted scans. **c,** Sedimentation coefficient distributions c(s) of each 0.5 mg ml^{-1} apoprotein and holoprotein at either 279, 340 or 437 nm, showing the relative abundances of predominant monomeric protein (~1 S) and nearby dimer. **d,** Absorption spectra of apoprotein and holoprotein taken from the first scan at central radial position during analytical ultracentrifugation as well as of NADPH and Pchlide in reaction buffer measured with a benchtop spectrometer. **e,** For better comparison, the data shown in d was scaled to yield similar absorption at the Pchlide Q_y-band. Scaling factors are given in the Figure. **f,** Extracted absorption spectra of the three major populations in 0.5 mg ml^{-1} holoprotein to assess the *Te*LPOR/NADPH/Pchlide content. The respective sedimentation coefficient ranges were selected from the peak borders as recognizable in panel c: monomer (0.47-1.50 S), dimer (1.50-2.20 S) and soluble Pchlide/NADPH aggregates (> 2.20 S). **g,** Sedimentation coefficient distributions c(s) of a 0.5 mg ml^{-1} holoprotein sample after illumination at either 340, 630 or 670 nm, showing the relative abundances of the monomeric protein (~1 S) and the nearby dimer. **h,** Extracted absorption spectra of the monomer and dimer populations of a 1.0 mg ml^{-1} sample of the illuminated holoprotein. The respective sedimentation coefficient ranges were selected from the peak borders as recognizable in panel h: monomer (0.32-1.2 S), dimer (1.2-2.03 S). To increase Pchlide turnover (Supplementary Fig. 2b), 0.03% (v/v) Triton X-100 was added to the protein solution before illumination.

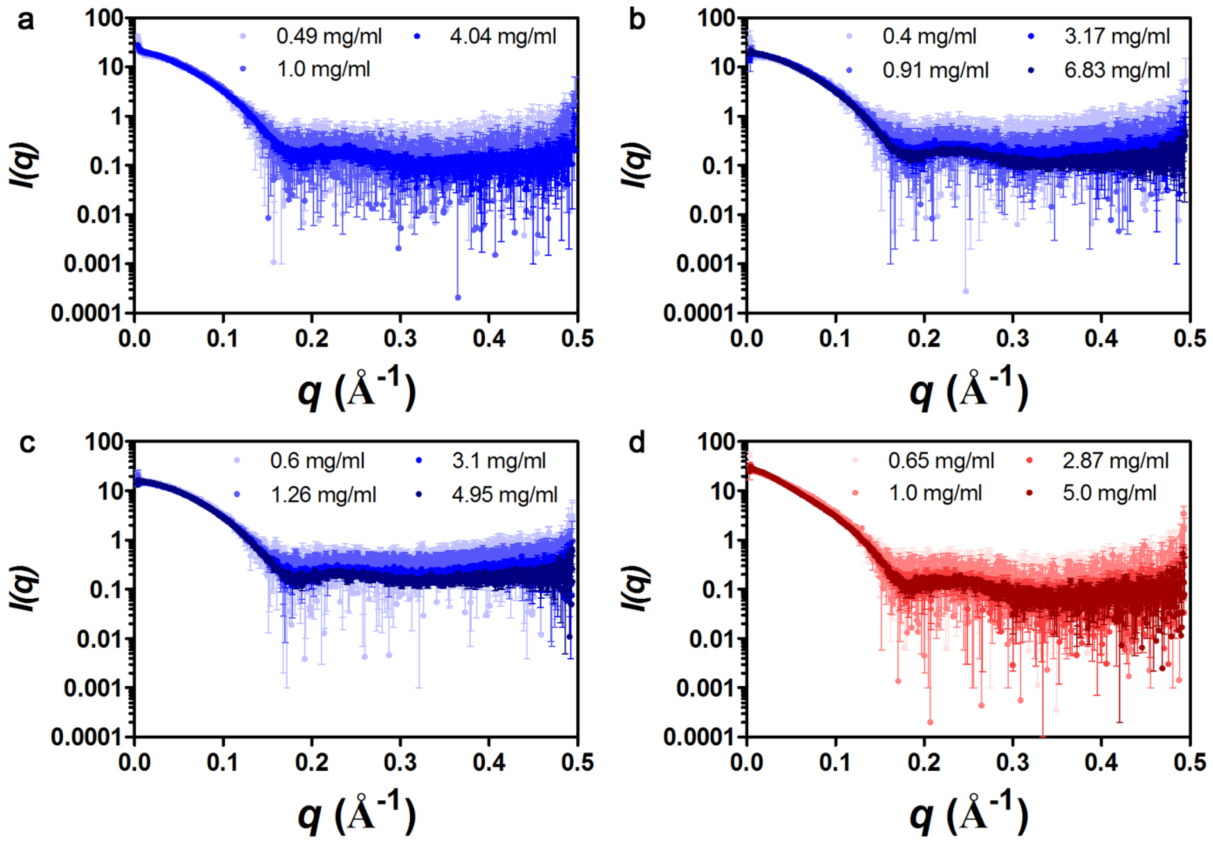

Supplementary Figure 4: a-c, SAXS scattering curves for three independently prepared and measured *Te*LPOR apoprotein samples and **d,** one *Te*LPOR holoprotein sample. For each sample SAXS data was acquired for a concentrations series of the indicated concentrations.

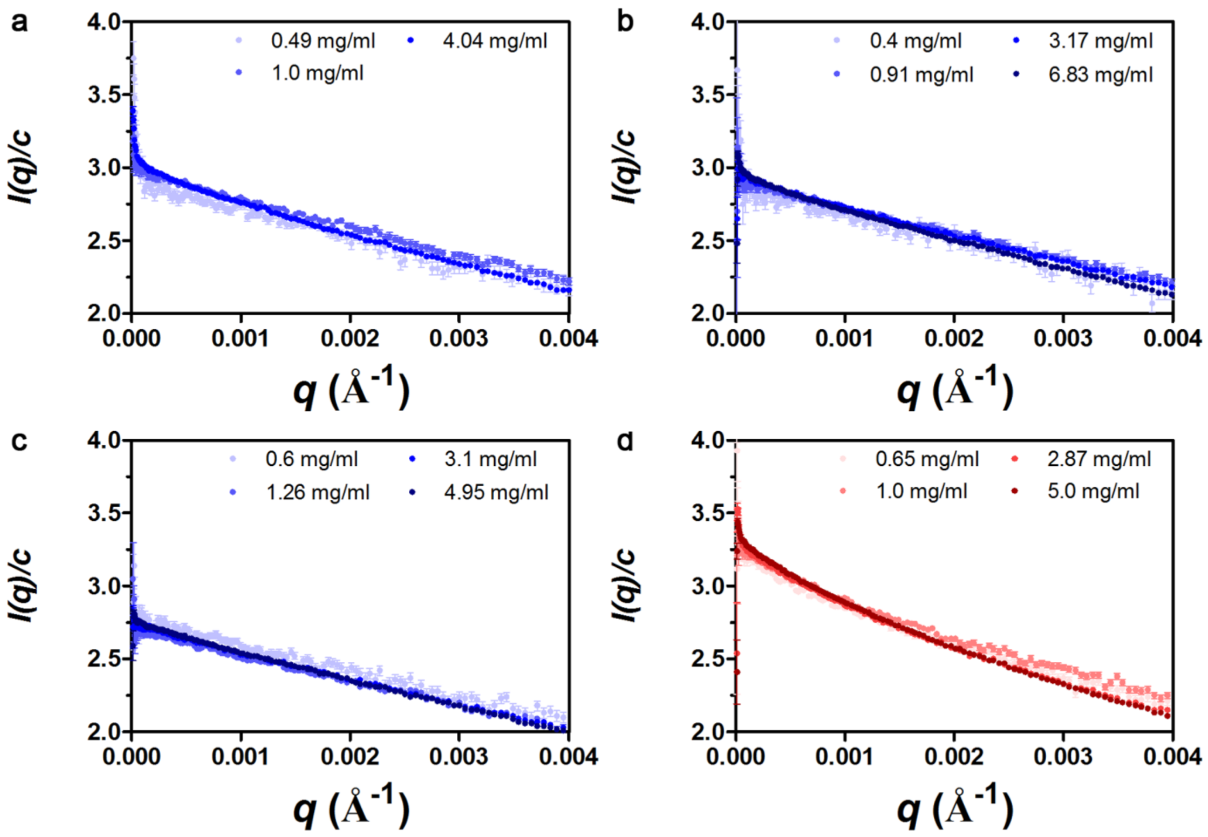

Supplementary Figure 5: a-c, Guinier plot (ln(*I(q)*) versus q^2) of the corresponding SAXS data at low *q* values for three independently prepared and measured *Te*LPOR apoprotein samples and **d,** one *Te*LPOR holoprotein sample. For each sample SAXS data was acquired for a concentrations series of the indicated concentrations.

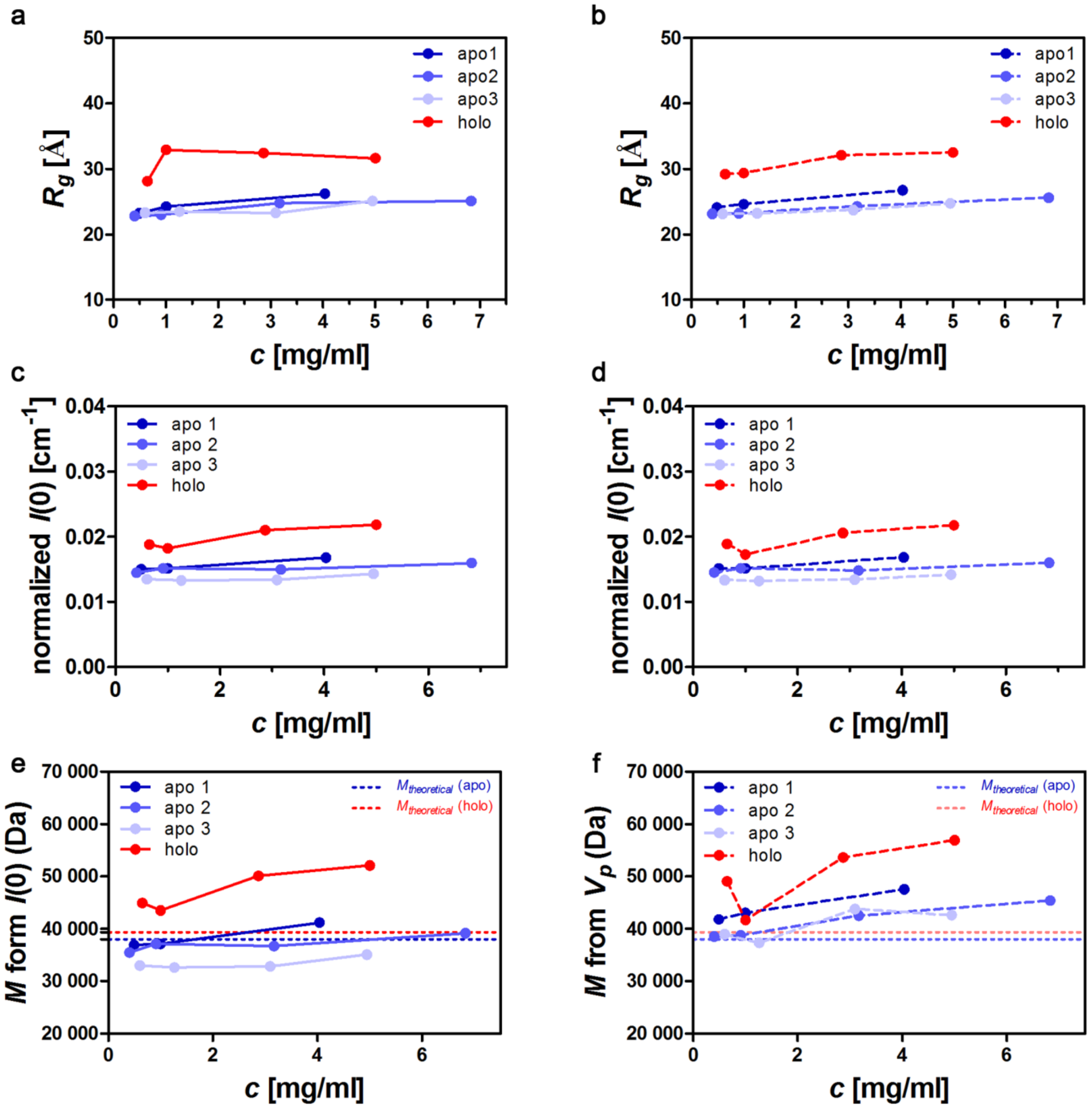

Supplementary Figure 6: Concentration dependence of the radius of gyration (R_g) and the forward scattering intensity $I(0)$ from **a,c,** Guinier and **b,d** distance distribution analysis. Three independently prepared and measured *Te*LPOR apo- (in shades of blue as indicated), and one *Te*LPOR holoprotein sample (in red) were analyzed. Molecular mass of the scattering particle obtained from **e,** the forward scattering intensity $I(0)$ and **f,** the Porod volume V_p (F). The dashed lines mark the theoretical molecular mass of the apo- (in blue) and holoprotein (in red) monomer.

a

```
                   10        20        30        40        50        60        70
                    |         |         |         |         |         |         |
TeLPOR     MSDQPRPTVIITGASSGVGLYATKALANRGWHVIMACRNLEKAEQAAKNLQIPPEAYTILHLDLSSLASV
DPM        cccccccceeeccctcccccchhhhhhthccceehhhhhhhhhhhhhhccccccccccehchchcchhce
DSC        ccccccceeeeeccccccccchhhhhhccccceehhhhhhhhhhhhhhhccccccccceeeccccccccc
GOR1       etcccceeeeeeeeccceeeeehhhhhhhhheeeehhhhhhhhhhhhhheeeechhheeehhhhhhhhee
GOR3       ccccccceeeeeececcceeehehhhhhcchhehhhhhhhhhhhhhhhhhhccchhhhhhhhhhcchhee
MLRC       ccccccceeeeeecccchhhhhhhhhhhccceeeeehchhhhhhhhhhhccccccceeeeeechhhhhhh
PHD        ccccccceeeeeecccccchhhhhhhhhhccceeeehhhhhhhhhhhhhhccccccceeeeeccccchhhh
Predator   ccccccceeeeeecccccceeehhhhhhcccceeeehhhhhhhhhhhhhhhcccccceeeeeccccccccc
SOPM       eccccccceeeeeecccccceeehhhhhhtttceeeeehhhhhhhhhhhhhcccctcceeeeeechhhhhhh
Sec.Cons.  ccccccceeeeeecccccceeehhhhhhhccceeeehhhhhhhhhhhhhhccccccceeeee?chcchh??

                   80        90       100       110       120       130       140
                    |         |         |         |         |         |         |
TeLPOR     RGFVESFRALNRPLRALVCNAAVYYPLLKEPIYSVDGYEITVATNHLGHFLLINLLLEDLKNSPESDKRL
DPM        hceehhhhhhchchhhhhhhhhheccccccccccccccccheeehcccccchhhehhhhhhhcctctttthhh
DSC        cchhhhhhhhhhhhhhhhcccccccccccccccccccceeeeehhhcchhhhhhhhhhhcccccccccce
GOR1       eeeeeehhhhcchhheeeeeeeeeeeeeeeeeeeeeeeeeeehhhhhhhhheehhhhhhhhhcchhhhhee
GOR3       eeeehhhhhhcchhhhhehchhhcccccccceeecccceeeehhhhhhhhhhhhhhhhhhcccchhhhhe
MLRC       hhhhhhhhhhhhhhhhhhhhhhhhhhcccccceeecccceeeeecchhhhhhhhhhhhhhhhcccccccccee
PHD        hhhhhhhhhhhhhhhhhhhhhhhhhhhccccceeecceeeeeechhhhhhhhhhhhhhhhcccccccccee
Predator   chhhhhhhhcccccccccccceeeeccccccccccccceeeeeecccccchhhhhhhhhhhcccccccccee
SOPM       hhhhhhhhhhccchhhhhhhhhhhhhhhcccceeeettceeeeehcccchhhhhhhhhhhhtcccccccee
Sec.Cons.  hhhhhhhhhhc?hhhhhhhhhhh?ccccccc?eecccceeeee??h??hhhhhhhhhhhhcccccccccee

                  150       160       170       180       190       200       210
                    |         |         |         |         |         |         |
TeLPOR     VILGTVTANRKELGGKIPIPAPPDLGNLEGFEKGFKKPIAMINGKPFKSGKAYKDSKLCNMLTARELHRR
DPM        eeeceechhhcctccccccccccccccccccctcccccchhhccctcttcccctctcchcchhhhhhhhhh
DSC        eeeeccchhhhhccccccccccccccchhhhhhhccccceeeccccccccccccchhhhhhhhhhhhhhh
GOR1       eeeeeeehhhhttteeeeeeeectcehhhhhhhhhhhhheeeccctchhhhhhhhhhhhhhhhhhhhhhh
GOR3       eeeehhehchhhhcccccccccccccchhhhhhhhhchhhhhhcccccccccccchhhhhhhhhhhhhhh
MLRC       eeeeeeeccccccccccccccccccccccchhhcccceeeeeccccccccccccchhhhhhhhhhhhhhh
PHD        eeeeecccchhhhcccccccccccccccchhhhccccceeeeeccccccccccccchhhhhhhhhhhhhh
Predator   eeeecccccccccccccccccccccccccccccccccccccccccccccccccccchhhhhhhhhhhhhh
SOPM       eeeeeechtccctticcccccccccccchhhhhtccccceeeettcccctticcccchhhhhhhhhhhhhhhh
Sec.Cons.  eeeeeechch???cccccccccccccc??hhhhccccc?eeeccccccccccccchhhhhhhhhhhhhhh

                  220       230       240       250       260       270       280
                    |         |         |         |         |         |         |
TeLPOR     FHESTGIVFNSLYPGCVADTPLFRHHFPLFQKLFPLFQKKITGGYVSQELAGERVAMVVADPEFRQSGVH
DPM        hhhccceeecccccccccccccchccccchhhhhhchhhhcccccceechhhhchhhhheehcchhccctec
DSC        hhcccceeeccccccccccccccccccchhhhhhhhhhccccccccchhhhhhhhhheecccccccccce
GOR1       hhctttteeeeetcttteeeeehhhhhhchhhhhhhhhhhheettttceeeehhhhhhhhhhhhhhhhttccc
GOR3       ehccceeeeeccccccccccchhhhhchhhhhhhhhhhhhcccceeeechhhhhhhheeccchhheeeee
MLRC       hhhccceeeccccccccccccccccccchhhhhhhhhhhhhccccceh hhhcccceeeeeecccccccccc
PHD        hhcccceeeeecccccecccccccchhhhhhhhhhhhhhhhhcccchhhhhhccceeeeeeccccccccee
Predator   hhhcccccccccccccccccccccccccccccccccccccccccceeeecccccceeeeecccccccccc
SOPM       hhhttceeeetccccccccccccttcchhhhhhhhhhhhcttceehhhhttcheeeeeeccccccccce
Sec.Cons.  hh?ccceee?ccccccccccccccccc?hhhhhhhhhhhhccccce?hhhhc?hh??eeeccccccccc?

                  290       300       310       320
                    |         |         |         |
TeLPOR     WSWGNRQKEGRKAFVQELSAEASDEQKARRLWELSEKLVGLA
DPM        ccctcctccchhhhhhhhhhhhthhhhhhhhhhhhhhhheccc
DSC        eecccccchhhhhhhhhhhhhhhhhhhhhhhhhhhhhhhhccc
GOR1       cctcccccttthhhhhhhhhhhhhhhhhhhhhhhhhhhhhhh
GOR3       eeecccchhchhhhhhhhhhhhhhhhhhhhhhhhhhhhhhhh
MLRC       ccccccchhhhhhhhhhhhhhcchhhhhhhhhhhhhhhhhccc
PHD        eeeccchhhhhhhhhhhhhhhhhhhhhhhhhhhhhhhhhccc
Predator   ccccccchhhhhhhhhhhhhhhhhhhhhhhhhhhhhhhcccc
SOPM       eecccccchtthhhhhhhhhhhhhhhhhhhhhhhhhhhhhhhh
Sec.Cons.  ??ccccchhhhhhhhhhhhhhhhhhhhhhhhhhhhhhhhccc
```

b

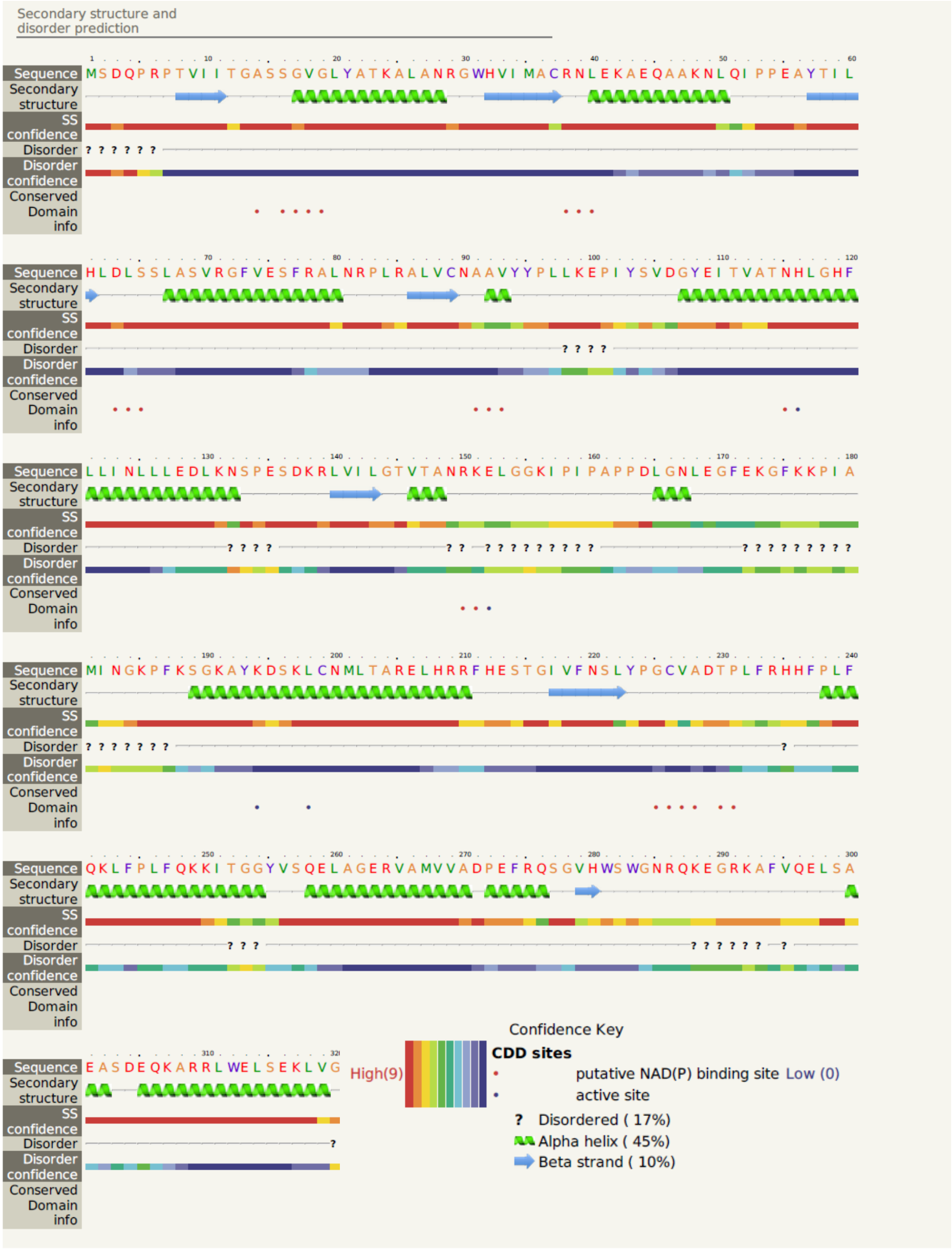

Supplementary Figure 7: *Te*LPOR Secondary structure prediction. **a,** The secondary structure of the *Te*LPOR was predicted using the NPS@ consensus secondary structure prediction webserver (https://npsa-prabi.ibcp.fr/cgi-bin/npsa_automat.pl?page=/NPSA/npsa_seccons.html) [1], that utilizes a variety of different algorithms for secondary structure prediction based on amino acid sequences. The secondary structure elements are given as α-helices (h), β-sheets (e), turn elements (t), random coils (c). **b,** Phyre2 [2] secondary structure prediction report.

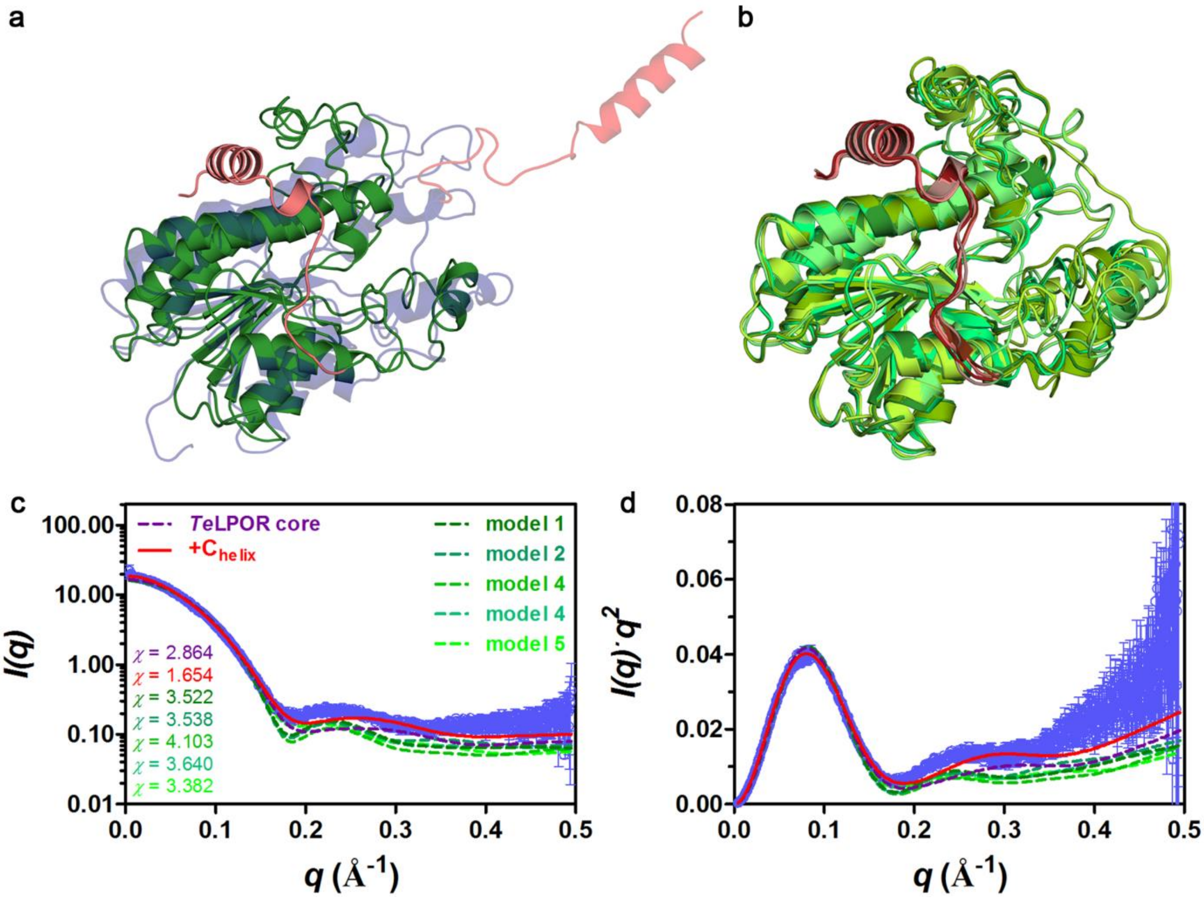

Supplementary Figure 8: I-TASSER-generated homology models and their CRYSOL-based evaluation. **a,** Superimposition of a representative I-TASSER-generated *Te*LPOR homology model (green, cartoon representation) in comparison to the C-terminally extended *Te*LPOR-C_{helix} model (blue, transparent cartoon). In both cases, the C-terminal extension adopts α-helical conformation (salmon, cartoon representation), either protruding from the *Te*LPOR core domain (*Te*LPOR-C_{helix}) or packing against the core domain Rossmann-fold (I-TASSER model). **b,** Superimposition of five I-TASSER-generated *Te*LPOR models (shades of green, cartoon representation). The C-terminal extension is shown as red cartoon. **c,d,** CRYSOL-based evaluation of the five I-TASSER generated homology models, showing **c,** SAXS scattering curve and **d,** the corresponding Kratky plot ($I(q) \cdot q^2$ versus q). The CRYSOL-derived theoretical scattering curve of the respective models (shades of green, dashed lines) was fitted to the experimental scattering data of the apoprotein (light blue, open circles). For comparison the CRYSOL-derived theoretical scattering curve of the *Te*LPOR core domain (pink dashed line) and of the *Te*LPOR-C_{helix} model (red solid line) is shown. No constant was subtracted. Data identity as indicated by the color code in panel c.

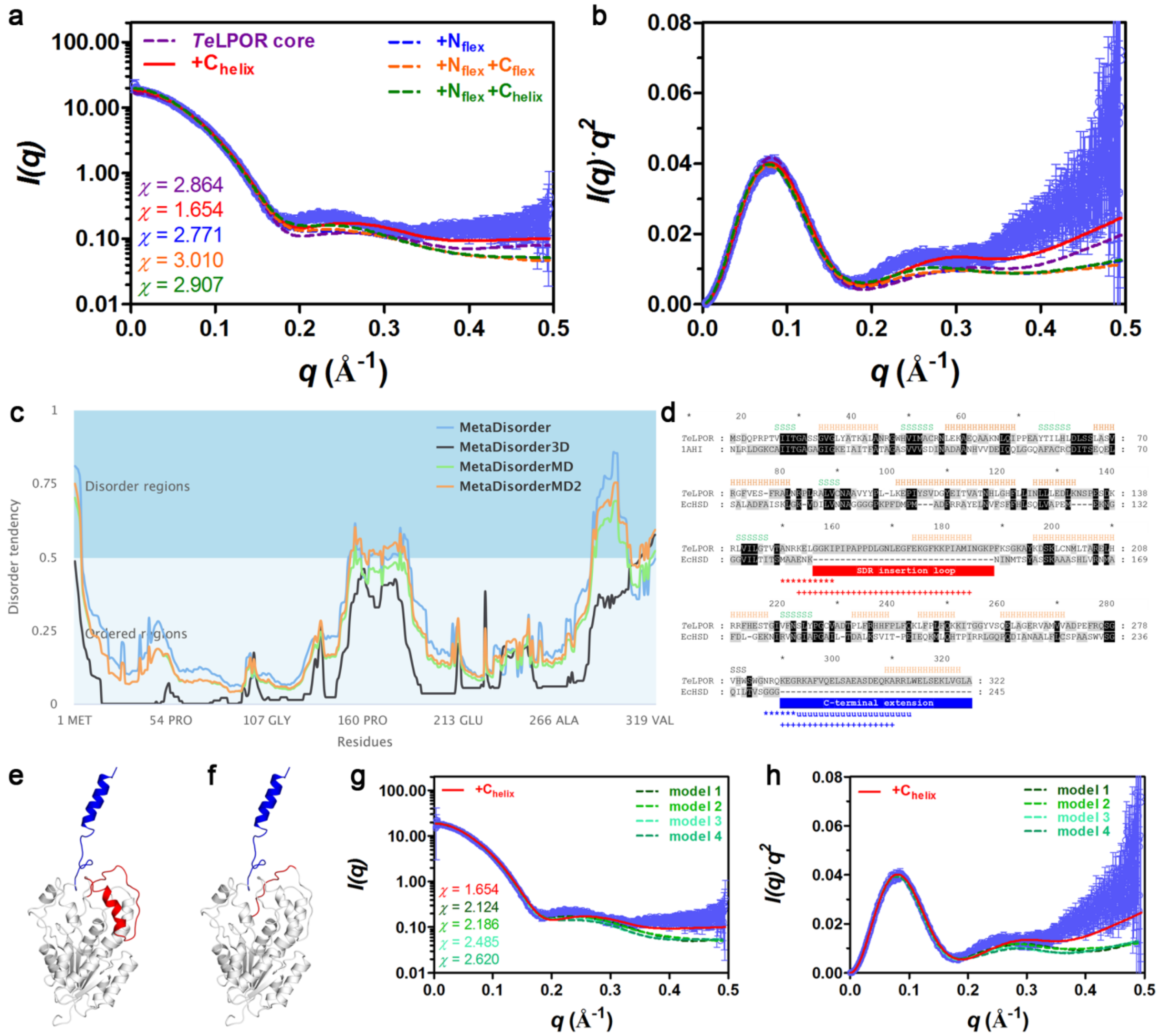

Supplementary Figure 9: EOM/CRYSOL-based evaluation of differently extended *Te*LPOR homology models. **a,** SAXS scattering curve. **b,** Kratky plot ($I(q){\cdot}q^2$ versus q). The EOM/CRYSOL-derived theoretical scattering curve of the respective model (dashed and solid lines) was fitted to the experimental scattering data of the apoprotein (light blue, open circles). No constant was subtracted. Overall five different models were compared: i) the *Te*LPOR core domain model (*Te*LPOR, dashed, dashed pink line) ii) the C-terminally extended model in which the missing C-terminal residues are modelled as protruding α-helix (+C_{helix}, solid red line), iii) *Te*LPOR core domain model extended by modelling the N-terminal His_6-tag as flexible ensemble with EOM (+N_{flex}, dashed blue line), iv) the C-terminally extended model completed by modelling the N-terminal His_6-tag as flexible ensemble N_{flex}+C_{helix}, dashed orange line), and v) the *Te*LPOR core domain model completed N- and C-terminally by modelling the missing residues as flexible ensemble (N_{flex}+C_{flex}, dashed green line). **c,** Disorder predictions for *Te*LPOR using the MetaDisorder webserver [3] revealed potential disordered regions for the N-terminus, the C-terminal extension (residue 285 onwards) and the LPOR insertion loop (residues 160 to 185). Amino acid sequence alignment of *Te*LPOR and a related 7α-hydroxysteroid dehydrogenase from *E. coli* (EcHSD, PDB-ID: 1AHI, [4]). Secondary structure, obtained from the *Te*LPOR homology model, assigned as α-helix (H) and β-strand (S) in orange and green, respectively. The LPOR insertion loop as well as the C-terminal extension, which are not present in related 7α-hydroxysteroid dehydogenases [5] are highlighted with red and blue boxes below the alignment. Stars mark flexible regions identified by NMR relaxation experiments of Armstrong [6]. Additionally, the C-terminal

region that could not be assigned in the NMR experiments (marked with u) [6], is highlighted. Potentially disordered regions as predicted by the Metadisorder webserver [3], which utilizes 13 primary disorder prediction programs, are marked by a plus sign (+) below the alignment. **e,f,** Illustration of the structural regions that were modelled as flexible/disordered ensemble, showing the C-terminal extension (highlighted in blue) as well as e, the whole insertion loop segment as defined by Townley [5] and f, a shorter segment of the insertion loop identified by NMR relaxation experiments as more mobile [6]. EOM/CRYSOL-based evaluation of different *Te*LPOR models (shades of green, dashed lines) possessing a flexible/disordered (model 1 and model 3) or rigid C-terminal extension (model 2 and model 4) as well as a completely flexible/disordered insertion loop (model 1) or a short flexible/disordered insertion loop (model 2). **g,** SAXS scattering curve. **h,** Kratky plot ($I(q) \cdot q^2$ versus q). The EOM/CRYSOL-derived theoretical scattering curve of the respective model (dashed and solid lines) was fitted to the experimental scattering data of the apoprotein (light blue, open circles). No constant was subtracted. For comparison the CRYSOL-derived theoretical scattering curve of the TeLPOR+C_{helix} model (solid red line) is shown.

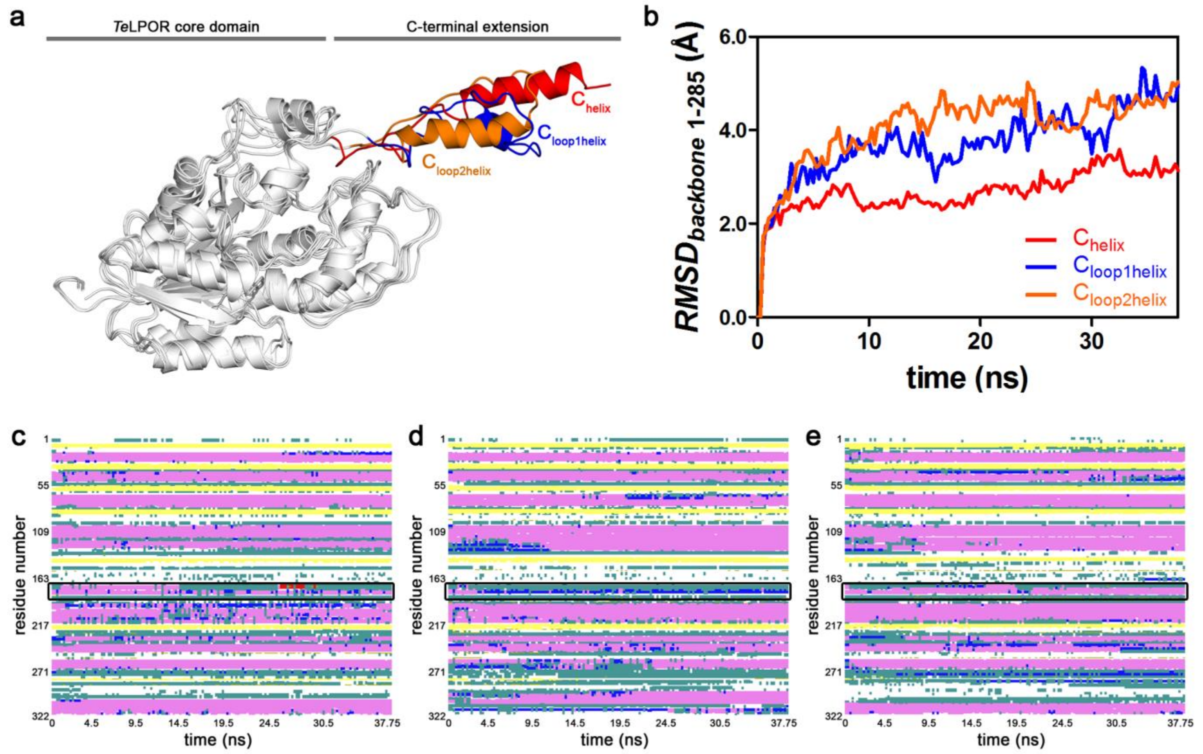

Supplementary Figure 10: a, MD simulations of *Te*LPOR homology models with different C-terminal extensions. Three different models (*Te*LPOR-C_{helix} (red), *Te*LPOR-$C_{loop1helix}$ (blue) and *Te*LPOR-$C_{loop2helix}$ (orange) were generated possessing different protruding C-terminal extensions. **b,** *Te*LPOR core domain stability inferred from the simulation trajectories of *Te*LPOR-C_{helix} (red line), *Te*LPOR-$C_{loop1helix}$ (blue line) and *Te*LPOR-$C_{loop2helix}$ (orange line). Root mean square deviation (RMSD) of the *Te*LPOR core domain (residues 1 to 285) plotted against the simulation time. Trajectories were superimposed over the *Te*LPOR core domain (residue 1 to 285) backbone atoms, yielding average RMSD values of 2.65 (*Te*LPOR-C_{helix}), 3.71 (*Te*LPOR-$C_{loop1helix}$) and 4.1 (*Te*LPOR-$C_{loop2helix}$). **c-e,** Secondary-structure stability over the simulation trajectories of *Te*LPOR-C_{helix} (c), *Te*LPOR-$C_{loop1helix}$ (d) and *Te*LPOR-$C_{loop2helix}$ (e). Secondary structure information was obtainded with VMD using STRIDE. Color code: turn-elements (dark cyan), extended β-sheets (light yellow), isolated β-bridge (dark yellow), α-helix (dark pink), 3-10 helix (light pink), Pi-helix (red) and random coil (white). The black boxes mark the position of the LPOR insertion loop (see also Supplementary Fig. 9c-f), which is not present in other short-chain dehydrogenases [5].

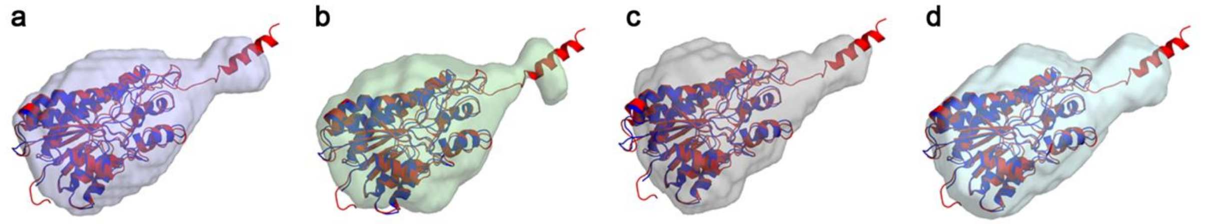

Supplementary Figure 11: *Ab initio* bead models of the *Te*LPOR apoprotein. Depicted is the SITUS derived envelope function of the DAMAVER-generated averaged and filtered models derived from 20 individual **a,** DAMMIF, **b,** DAMMIN and **c,** GASBORP runs. Superimposed on the respective envelope, the *Te*LPOR homology model of the core domain (blue) and the C-terminally extended *Te*LPOR-C_{helix} model (red) is shown. Fitting of the homology models to the respective envelope was achieved by using the tool COLORES of the SITUS package [7]. While for each envelope slightly different rotational orientations for the homology models are obtained by COLORES, the overall orientation in terms of model placement was very similar. For clarity, we therefore only show the best fit orientation obtained for the superimposition of the homology models and the DAMMIF envelope. The *ab initio* models shown in a-c were generated using the merged SAXS data of the apo 2 protein sample. **d,** SITUS derived envelope function of the DAMAVER-generated averaged and filtered model derived from 20 individual DAMMIF runs using a single low concentration SAXS dataset (apo 2 sample, 0.91 mg ml^{-1}).

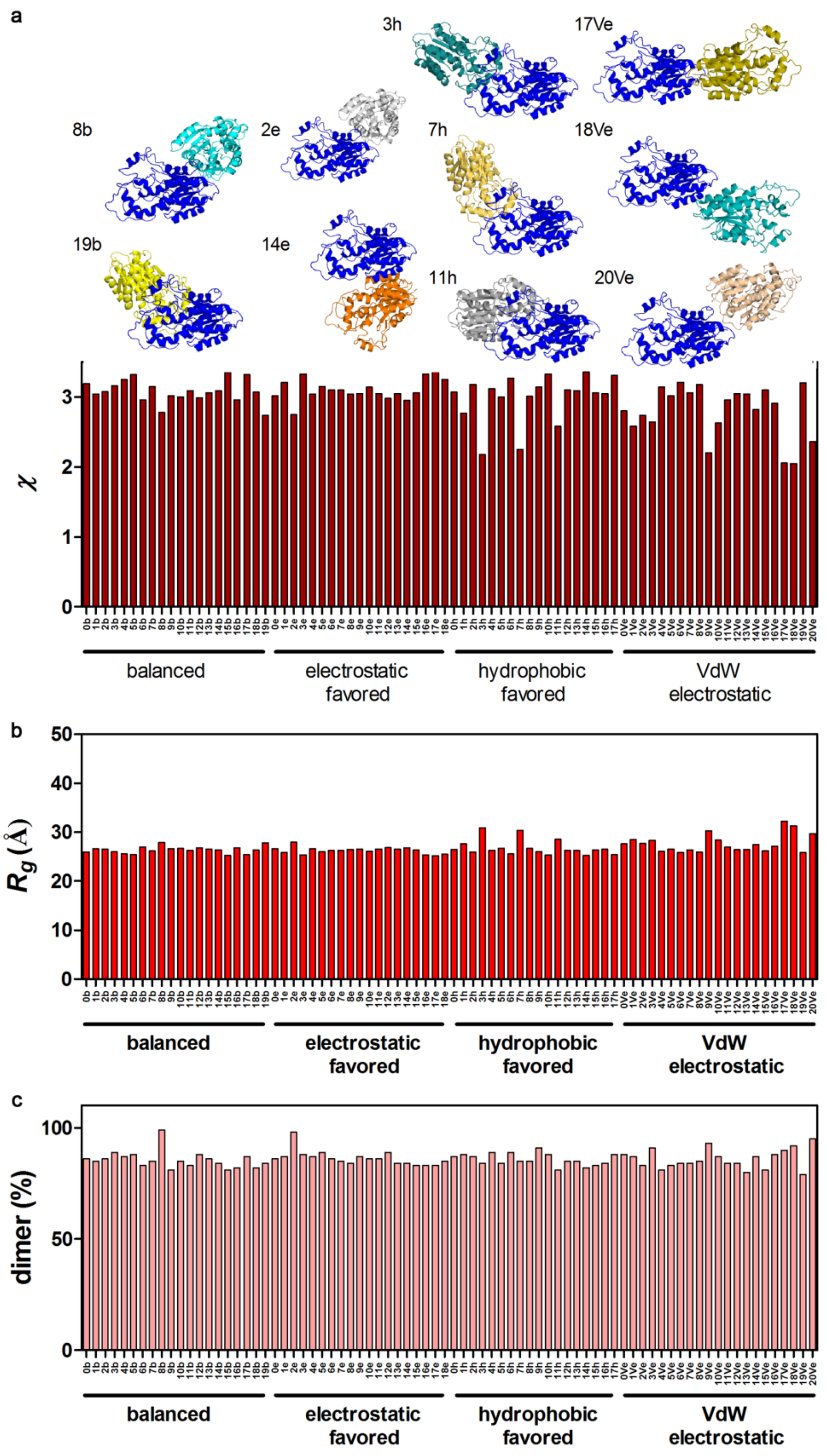

Supplementary Figure 12: *Te*LPOR ternary complex dimer modelling – core domain dimer models. Evaluation of *Te*LPOR dimer models in terms of **a,** χ **b,**radius of gyration (R_g) and **c,** dimer content. *Te*LPOR dimer models were generated by the ClusPro docking server in homo-multimer mode as described in the Supplementary Methods/Results section using the *Te*LPOR core domain model as monomer input model. All models were compared against the experimental SAXS scattering curve for the holoprotein sample at high concentration (5.0 mg ml^{-1}) using the program OLIGOMER [8]. Form factor files for the monomer and the corresponding dimer were generated from the respective pdb file using FFMAKER.

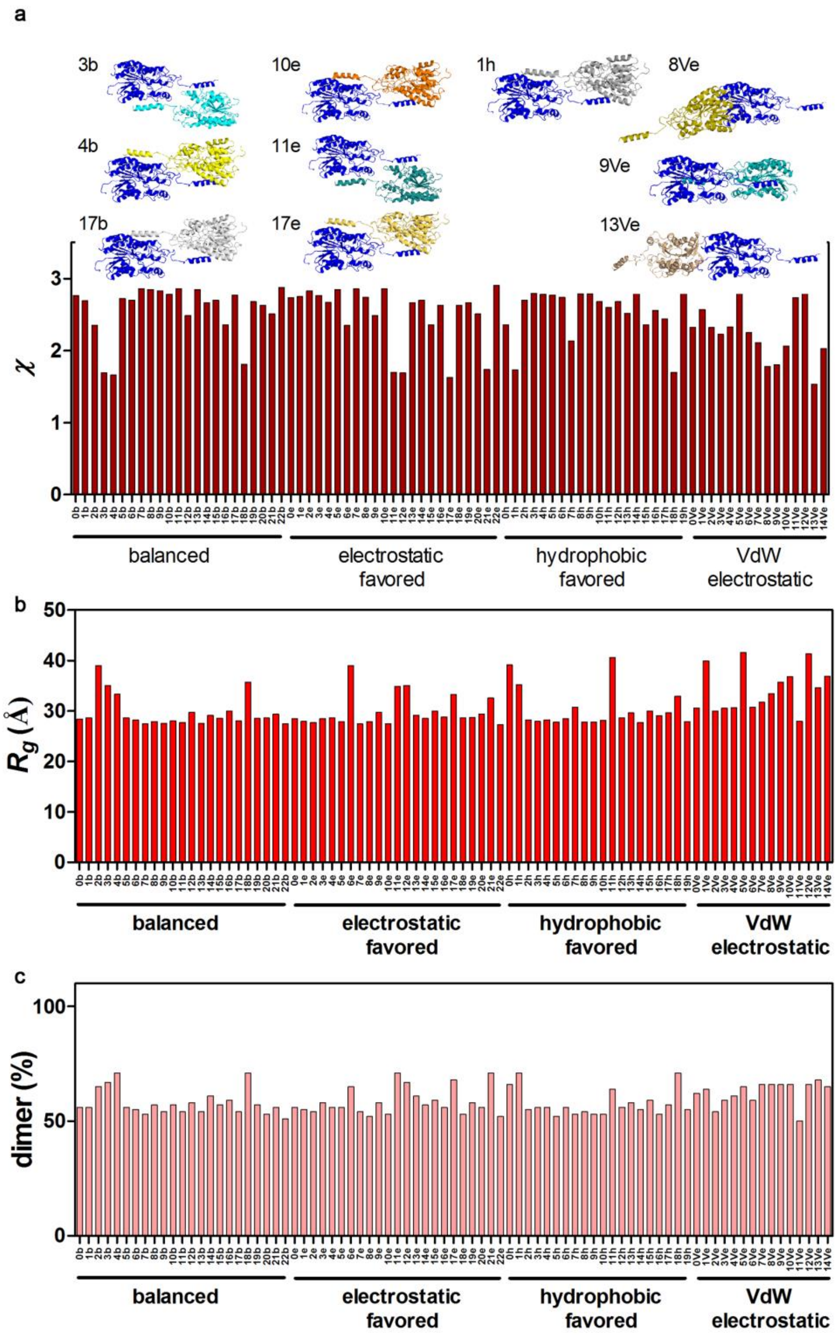

Supplementary Figure 13: *Te*LPOR ternary complex dimer modelling – C-terminally extended dimer models. Evaluation of *Te*LPOR dimer models in terms of **a,** χ **b,** radius of gyration (R_g) and **c,** dimer content. *Te*LPOR dimer models were generated by the ClusPro docking server in homo-multimer mode as described in the Supplementary Methods section using the C-terminally extended *Te*LPOR model as monomer input model. All models were compared against the experimental SAXS scattering curve for the holoprotein sample at high concentration (5.0 mg ml^{-1}) using the program OLIGOMER [8]. Form factor files for the monomer and the corresponding dimer were generated from the respective pdb file using FFMAKER.

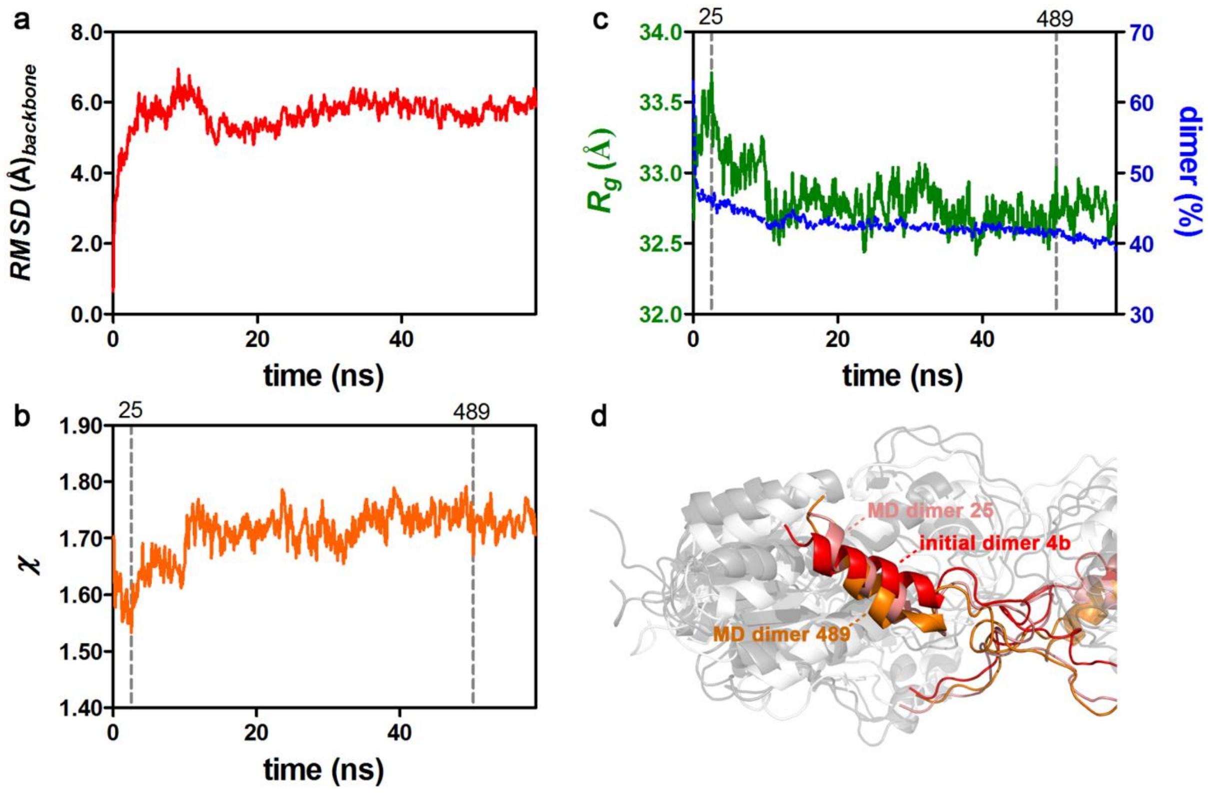

Supplementary Figure 14: MD simulation of the ternary complex holoprotein dimer model 4b. **a,** Root mean square reviation (RMSD) of the backbone atoms of the full length dimer (residues 1-322; starting coordinates as reference) plotted against the simulation time. **b,** Evaluation of MD trajectory snapshots in terms of χ plotted against the simulation time. From the 58.8 ns trajectory 583 snapshots were extracted (every 100 ps), which were individually compared against the experimental SAXS scattering curve for the holoprotein sample at high concentration (5.0 mg ml^{-1}) using the program OLIGOMER [8]. Form factor files for the monomer and the corresponding dimer were generated from the respective snap shot pdb file using FFMAKER. **c,** Radius of gyration (R_g)(green, lefit ordinate) and dimer content (blue, right ordinate) as obtained from the MD trajectory snapshots by OLIGOMER plotted against the simulation time. **d,** Superimposition of the starting structure (dimer model 4b), and selected MD trajectory snapshots . As representative snapshots, the model showing the best χ value for the fit against the experimental SAXS data (snapshot 25) and a late snapshot after convergence of the RMSD and χ value (snapshot 489) were selected. Both data points are marked by grey dashed lines in b and c.

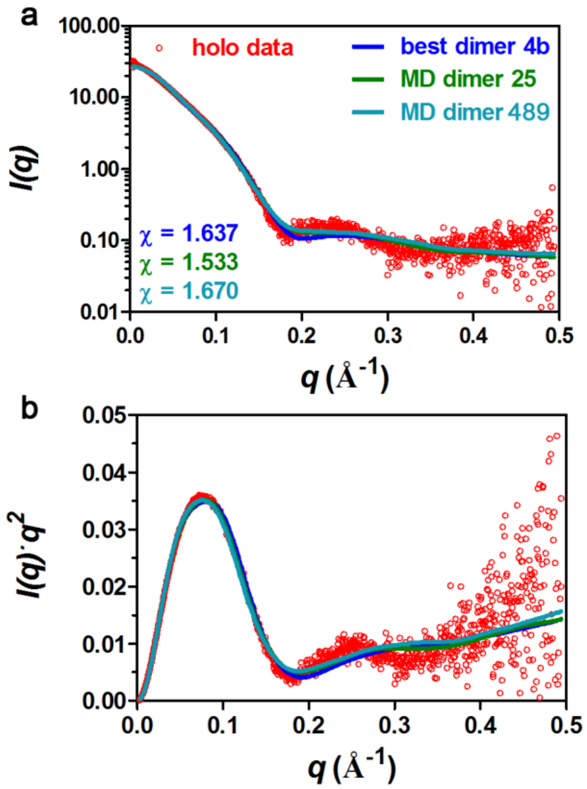

Supplementary Figure 15: a, Experimental scattering data and **b,** Kratky plot (*I(q)*·q^2 versus *q*) of the holoprotein (red, open circles) and the fit of the OLIGOMER-derived theoretical scattering curve of a monomer/dimer mixture for the initial dimer 4b (blue, solid line), an early MD snapshot (green, solid line, snapshot MD dimer 25) and a late MD snapshot extracted after convergence of RMSD (cyan, solid line, snapshot MD dimer 489). Form factor files for the monomer and the corresponding dimer were generated from the respective monomer and dimer pdb files using FFMAKER.

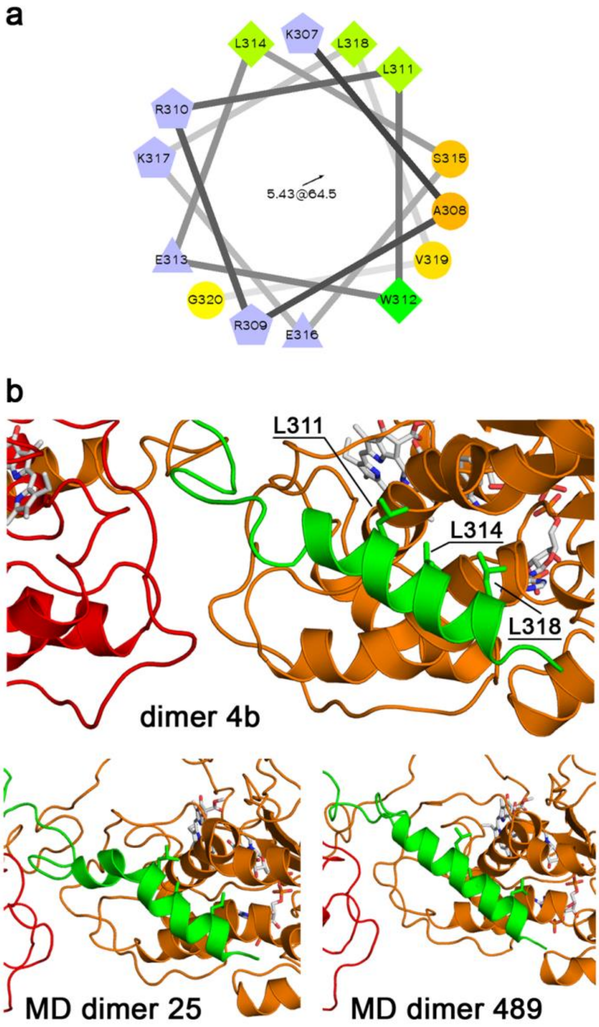

Supplementary Figure 16: a, Helical-wheel projection of the C-terminal extension and **b,** dimer subunit interaction via the C-terminal helical extension. The helical wheel was generated using the helical wheel plotter available via the Raphael Zidovetzki Lab website (http://rzlab.ucr.edu/scripts/wheel/wheel.cgi). Cartoon representation of the 4b dimer model, the MD dimer 25 and 489, with the two *Te*LPOR subunits shown in red and orange and the C-terminal extension of one subunit colored in green. Hydrophobic residues (L311, L314, L318) that are part of the hydrophobic side of the amphipathic C-terminal helix, are shown in stick representation.

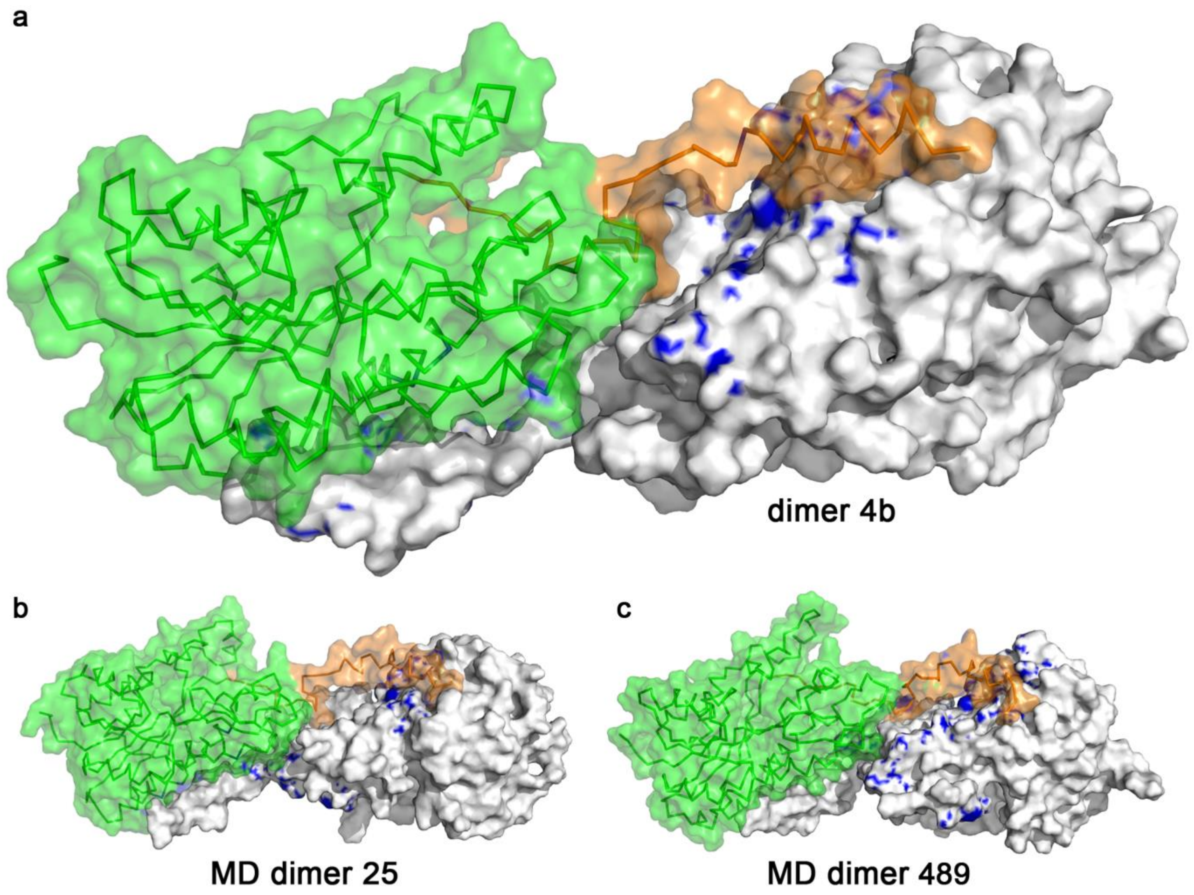

Supplementary Figure 17: a, Hydrophobic dimer interface patches of the ClusPro-derived dimer 4b, **b,** the MD-derived dimer 25 and **c,** the MD-derived dimer 489. Hydrophobic surface patches were identified using the hpatch tool of the Rosetta protein design software [9,10]. In each panel chain A of the dimer is shown as Cα-trace with green transparent surface, with the C-terminal extension (residue 287-322) highlighted in orange, respectively. Chain B of the dimer is shown as white solid surface. Hydrophobic surface patches are highlighted in blue on both chains. For clarity only hydrophobic surface patches at the dimer interface are depicted.

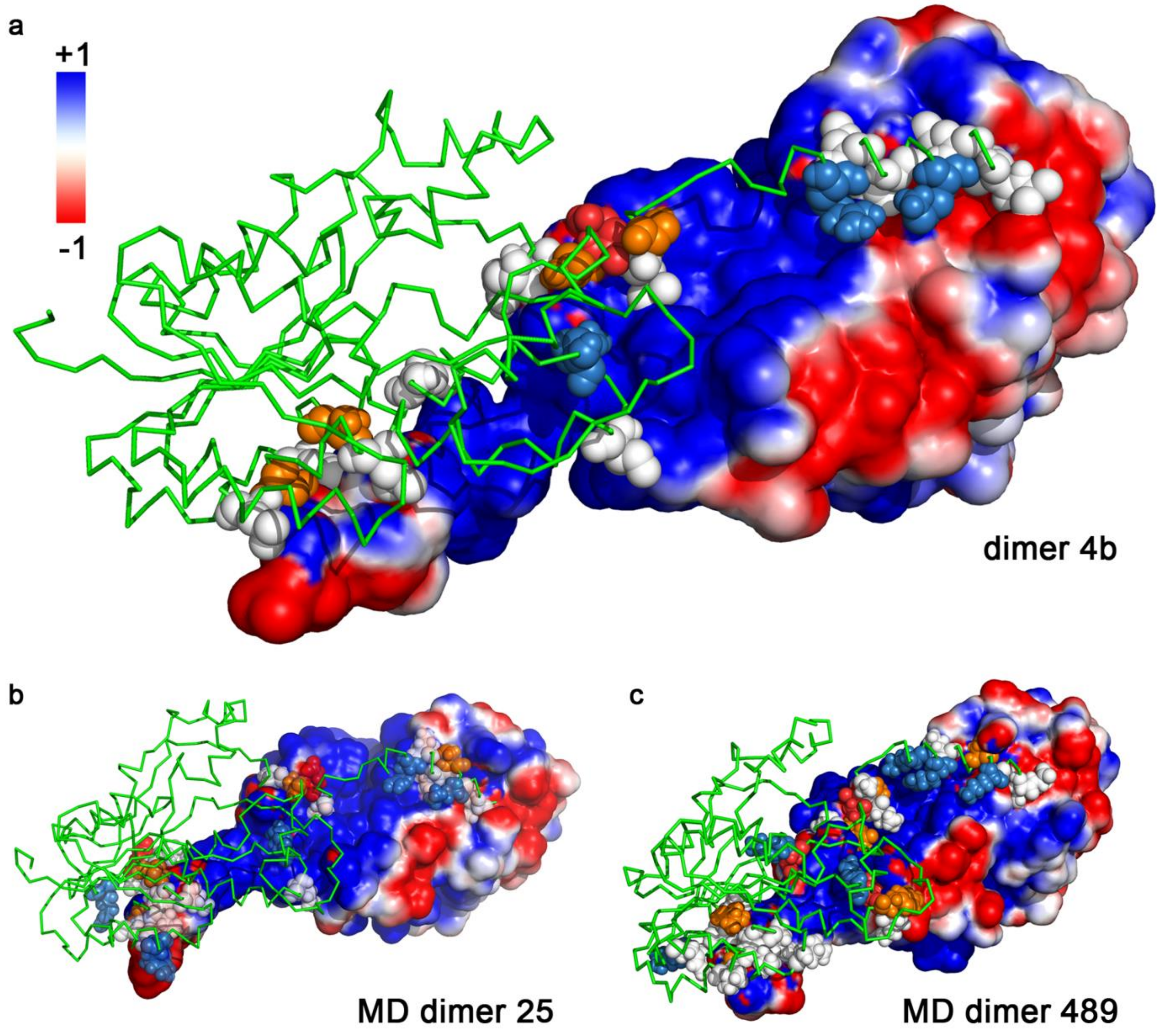

Supplementary Figure 18: a, Electrostatic surface potential of the ClusPro-derived dimer 4b, **b,** the MD-derived dimer 25 and **c,** the MD-derived dimer 489. In each panel, the structure of chain A of the dimer is shown as green ribbon, while chain B is shown as surface with mapped electrostatic potential. The potential computed is shown from -1.0 kcal/mol/e (red) to +1.0 kcal/mol/e (blue). For each model, interface residues were identified using the WHAT-IF web service (https://swift.cmbi.umcn.nl) and interface residues are shown as spheres with positively charged residues in blue, negatively charged residues in red, polar residues in orange and hydrophobic residues in white. The electrostatic calculations were performed based on the Poisson Boltzmann (PB) model [11]. The electrostatic potential maps were generated using by Pymol plugin for Adaptive Poisson Boltzmann Solver (APBS). Pymol was used to prepare molecular structures for electrostatic calculations.

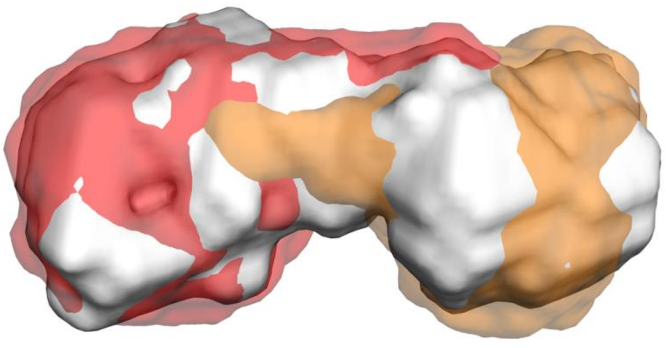

Supplementary Figure 19: Superimposition of two GASBORP-derived monomeric apoprotein envelopes (shown as red and orange transparent surface; contoured at 1.0 sigma) on the GASBORMX-derived dimeric holoprotein envelope (grey solid surface; contoured at 1.0 Sigma). Map in map fitting of the corresponding Situs maps was performed using UCSF Chimera 1.11.

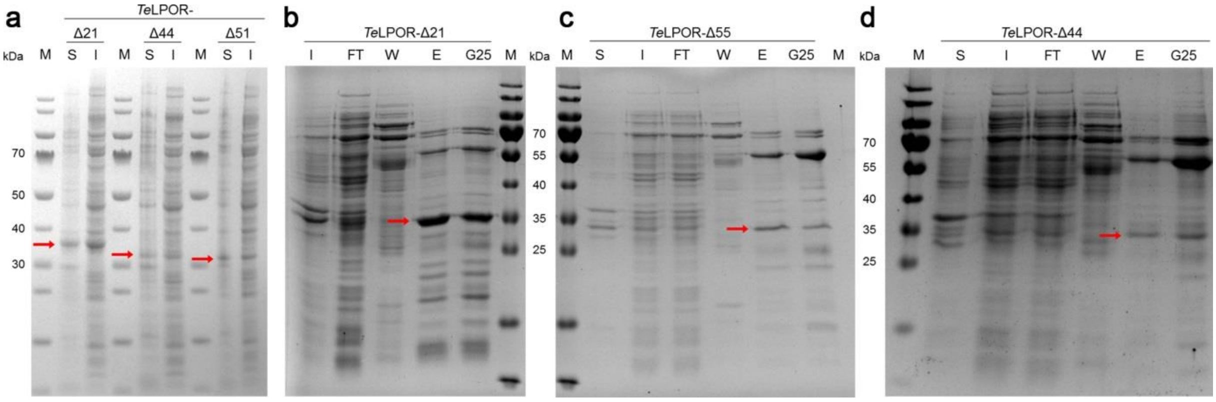

Supplementary Figure 20: SDS-PAGE analysis illustrating **a,** the heterologous production of the C-terminally truncated *Te*LPOR variants, and the IMAC purification of **b,** *Te*LPOR-Δ21, **c,** *Te*LPOR-Δ55 (B) and **d,** *Te*LPOR-Δ44 (C). In a, the soluble protein fraction (S) and the insoluble protein fraction (I) obtained by centrifugation of the corresponding crude cell extracts after cell lysis are shown. 10-12% pre-cast Bis-Tris gel, MES running buffer; In b-d, additionally the IMAC flow-through fraction (FT), the wash fraction (W), elution fraction (E) as well as the final sample after desalting (G25) is shown. 12% Tris-Glycine gel. The red arrow marks the expected molecular mass (*Te*LPOR-Δ55: 32,106 Da; *Te*LPOR-Δ44: 32,908 Da; *Te*LPOR-Δ21: 35,633 Da) of the respective target protein. The samples shown in b and c were run on the same gel. For clarity of illustration, the gel image was separated after the marker (b) or before the marker (c). M: PageRulerTM prestained protein ladder, 10-180 kDa (Thermo Fisher Scientific, Waltham, MA, USA)

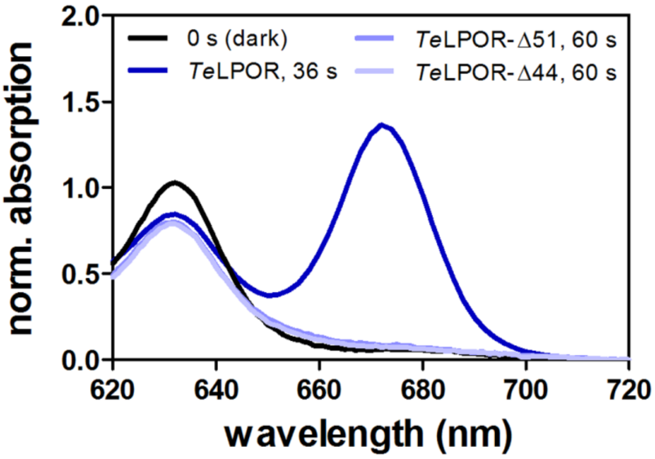

Supplementary Figure 21: Light dependent Pchlide turnover, analyzed using purified protein preparations of *Te*LPOR-Δ51, *Te*LPOR-Δ44 and *Te*LPOR wild type. All measurements were performed as described for the crude-cell extract samples (Figure 5f, main manuscript), with the exception of a shorter illumination time used for *Te*LPOR wildtype. Sample identity color coded as indicated.

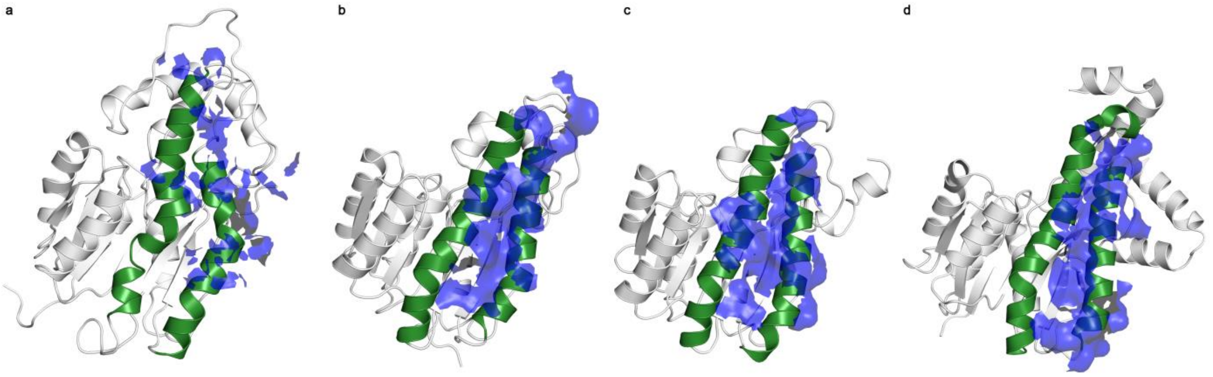

Supplementary Figure 22: Illustration of the Q-axis interface of short-chain dehydrogenases/reductases. Rossmann-fold core domain of **a,** *Te*LPOR, **b,** a 7α-hydroxysteroid dehydrogenase (HSD) of *E. coli*, **c,** a 3α,20β-HSD of *Streptomyces exfoliates* and **d,** human 17β-HSD. All structures shown in cartoon representation, with the two long α-helices (usually termed αE and αF), which constitute the Q-axis oligomerization interface highlighted in green. Hydrophobic surface patches were identified using the hpatch tool of the Rosetta protein design software [9,10]. The hpatch identified hydrophobic surface patches are shown as transparent blue surface. For clarity only hydrophobic surface patches at the oligomer interface are depicted. PDB-IDs: b, 1AHI; c, 2HSD, d, 1QYV

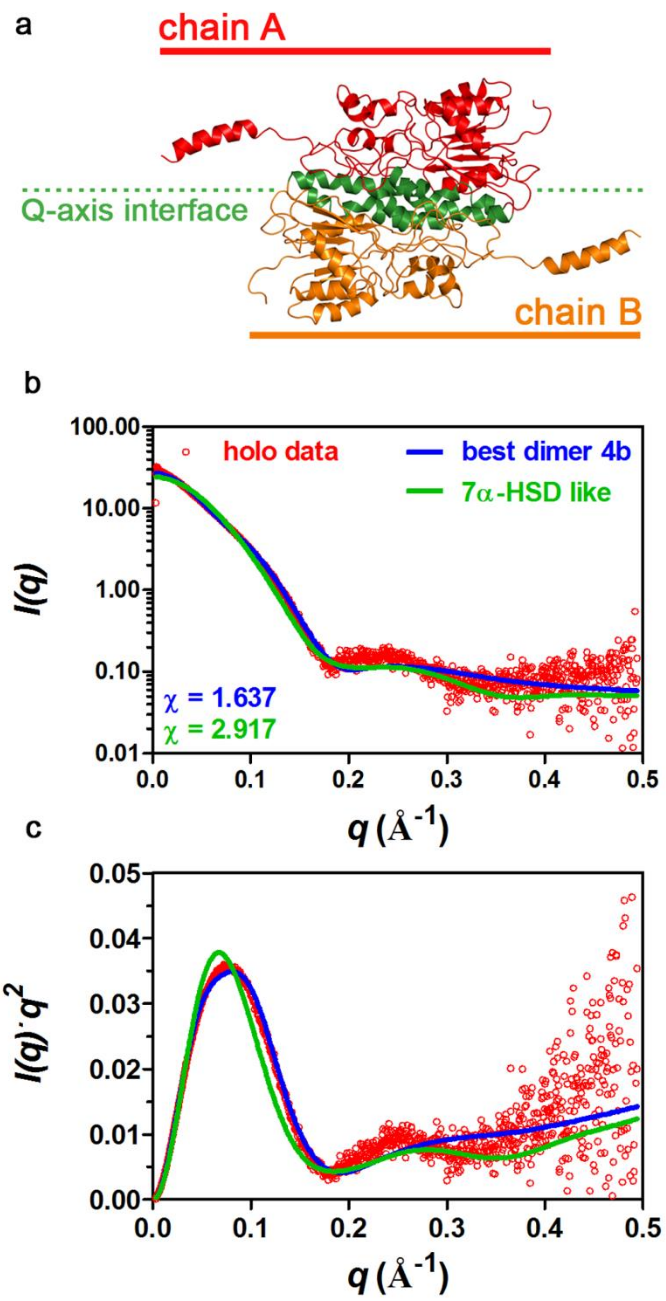

Supplementary Figure 23: Evaluation of a *Te*LPOR dimer model, dimerized via the Q-axis interface of 7α-hydroxysteroid dehydrogenases (HSDs). **a,** 7α-HSD-like *Te*LPOR dimer shown in cartoon representation, with the two dimer subunits colored in red and orange. The Q-axis dimer helices are highlighted in green on both subunits. The 7α-HSD-like *Te*LPOR dimer model was generated, by superimposing two molecules of the full-length *Te*LPOR monomer to the structure of a 7α-HSD from *E. coli* (PDB-ID: 1AHI). **b,** Experimental scattering data and **c,** Kratky plot ($I(q) \cdot q^2$ versus q) of the holoprotein (red, open circles) and fit of the OLIGOMER-derived theoretical scattering curve of a monomer/dimer mixture of the 7α-HSD-like *Te*LPOR dimer (green solid line) and the ClusPro-derived dimer 4b (blue, solid line) Form factor files for the monomer and the corresponding dimer were generated from the respective monomer and dimer pdb files using FFMAKER.

Supplementary Tables

Supplementary Table 1: Experimental details for SAXS data acquisition, evaluation and modelling

**a) Sample details**		
	*Te*LPOR apo 2	*Te*LPOR holo
Organism	*Thermosynechococcus elongatus* BP-1	*Thermosynechococcus elongatus* BP-1
UniProt ID (residues in construct)	recombinant protein, produced in *E.coli* BL21(DE3)	recombinant protein, produced in *E.coli* BL21(DE3)
Purification tag (residues/sequence)	N-terminal His6-tag (20 / MGSSHHHHHHSSGLVPRGSH)	N-terminal His6-tag (20 / MGSSHHHHHHSSGLVPRGSH)
Extinction coefficient ε (M^{-1} cm^{-1}) / A_{280} 0.1 % (*w/v*)	35,660 / 0.938	5,559; determined by taking into account the absorption contribution of Pchlide and NADPH at 280 nm
$\bar{\nu}$ from chemical composition (cm^3 g^{-1})	0.743	0.739
Particle contrast from sequence and solvent constituents, $\Delta\bar{\rho}$ ($\rho_{protein} - \rho_{solvent}$; 10^{10} cm^{-2})	2.11 (12.22-10.11)	2.15 (12.26-10.11)
M from chemical composition (Da)	38,014	39,370 (including Pchlide and NADPH)
Concentration series (mg ml^{-1}); by A_{280} *: merged<br>Injection volume (µl)<br>Flow rate	0.40 / 0.91* /3.17 / 6.83*<br>70µl<br>70µl/30s	0.65*/ 1.00 /2.87 / 5.00*<br>70µl<br>70µl/30s
Solvent (solvent blank; concentrator flow-through)	20 mM Tris pH 7.5, 500 mM NaCl, 20 % (w/v) glycerol	
**b) SAXS data collection**		
Instrument/data processing	European Synchrotron Radiation Facility (ESRF) SAXS beamline 29 with PILATUS 1M detector [12]	
Wavelength (Å)	0.9919	
Beam size (µm)	700 x 700 at sample	
Camera length (m)	2.867	
q range ($Å^{-1}$)	0.00326 - 0.49460	0.00308 - 0.49444
Absolute or relative scaling method	absolute scaling by comparison with scattering from pure H_2O	
Normalization	to transmitted intensity monitored with a diode integrated in the beamstop	
Monitoring of radiation damage	frame by frame comparison	
Exposure time (s) / no. of frames	3 /10	0.5 / 10
Sample configuration	concentration series of samples (see a) injected from automated sample changer; continuously purged through quartz capillary of 1.8 mm diameter	
Sample temperature (° C)	10	
**c) Software employed for SAXS data reduction, analysis and interpretation**		
SAXS data reduction	PRIMUSqt (r3709) / ATSAS 2.6.1 [13]	
Extrinction coefficient	ProtParam [14]	
Calculation of $\bar{\nu}$ and $\Delta\bar{\rho}$ values	MULCh 1.1 (29/10/17) [15]	
Basic analyses: Guinier, *P(r)*, V_P	PRIMUSqt (r3709) [8,13]	
Bead modelling	DAMMIF [16], DAMMIN [17]. GASBORP [18]	GASBORMX [18]
Atomic structure modelling	YASARA Version 16.6.24 [19,20], Crysol [21]	
Missing sequence modelling	YASARA 16.6.24 [19,20], EOM [22,23]	
Structure visualization	*PyMOL* v1.7.0.0 Win32; VMD v1.9.2 Win32 [24]	
**d) Structural parameters**		
**Guinier analysis**		
I(0) (cm^{-1})	0.013785 ± 0.00005	0.109217 ± 0.00037
R_g (Å)	23.0 ± 0.015	31.6 ± 0.013
q_{min} ($Å^{-1}$)	0.0165	0.0292
qR_g max	1.3	1.266
Coefficient of correlation, R^2	0.999	0.942
M from *I(0)* (Da) (ratio to predicted)	37,096 ± 131 (0.98)	52,108 ± 176 (1.32)
***P(r)* analysis**		
I(0) (cm-1)	0.013671 ± 0.00034	0.110305 ± 0.00019
R_g (Å)	22.9 ± 0.006	32.5 ± 0.006
D_{max} (Å)	80.5	105.4
q range ($Å^{-1}$)	0.0160 – 0.347	0.0282 – 0.254
χ^2 (total estimate from GNOM)	1.03 (0.636)	1.12 (0.555)
M from *I(0)* (Da) (ratio to predicted)	36,789 ± 87 (0.97)	52,627 ± 89 (1.33)
Porod volume ($Å^{-3}$)	65,730	96,680

M from Porod volume (ratio to predicted) (Da)	38,649 (1.02)	55,895 (1.42)
e) Envelope model-fitting results		
DAMMIF (default parameters, 20 models, slow mode)		
q range for fitting	0.0160 - 0.347	
Symmetry, anisotropy	P1	
NSD (standard deviation), number of clusters	0.497 (0.027), 1	
χ^2 range	1.07 - 1.13	
M estimate (Da) (ratio to predicted)	35,910 (0.94)	
DAMMIN (default parameters, 20 models)		
q range for fitting	0.0160 – 0.347	
Symmetry, anisotropy	P1	
NSD (standard deviation), number of clusters	0.519 (0.011), 1	
χ^2 range	1.08 - 1.10	
M estimate (Da) (ratio to predicted)	31,110 (0.82)	
GASBORP (default pararmeters, 20 models)		
q range for fitting	0.0160 - 0.347	
Symmetry, anisotropy	P1	
NSD (standard deviation), number of clusters	0.918 (0.025), 1	
χ range	1.83 - 2.15	
M estimate (Da) (ratio to predicted)	29,498 (0.77)	
GASBORMX		
q range for fitting		0.0282 – 0.254
Symmetry, anisotropy		P2
NSD (standard deviation) , number of clusters		1.197 (0.025), 1
χ^2 range		1.15 - 1.45
M estimate (Da) (ratio to predicted)		58,153 (1.48)

Supplementary Table 2: Evaluation of the SAXS scattering data for the *Te*LPOR apoprotein and the NADPH/Pchlide/TeLPOR ternary holoprotein complex

Sample	c (mg ml^{-1})	*I*(0) (cm^{-1})[a]	*M* from *I(0)* [a] (Da)	R_g (Å)	Monomer [%][c]	Porod volume (Å^3)	*M* from Porod volume[b]	Monomer [%][c]
apo1	0.49	0.007382 ± 0.00006	36,892 ± 301	23.2 ± 0.46	103.0 ± 0.8	71.110	41.813	90.0
	1.0	0.015148 ± 0.00005	37,096 ± 133	24.2 ± 0.18	102.4 ± 0.4	73.130	43.000	86.9
	4.04	0.067961 ± 0.00012	41,196 ± 74	26.2 ± 0.09	91.6 ± 0.2	80.910	47.575	74.8
apo2	0.4	0.005791 ± 0.00005	35,457 ± 301	22.8 ± 0.44	106.7 ± 0.8	65.510	38.520	98.7
	0.91[*]	0.013785 ± 0.00005	37,096 ± 131	23.0 ± 0.15	102.4 ± 0.4	65.860	38.726	98.1
	3.17	0.047489 ± 0.00009	36,687 ± 70	24.8 ± 0.12	103.5 ± 0.2	72.330	42.530	88.1
	6.83[*]	0.109178 ± 0.00019	39,146 ± 70	25.1 ± 0.08	97.0 ± 0.2	77.190	45.388	80.6
apo3	0.6	0.008085 ± 0.00005	32,997 ± 193	23.3 ± 0.26	113.2 ± 0.6	66.190	38.920	97.6
	1.26	0.016767 ± 0.00005	32,588 ± 100	23.5 ± 0.2	114.3 ± 0.3	63.470	37.320	101.8
	3.1	0.041511 ± 0.00006	32,792 ± 45	23.3 ± 0.2	113.7 ± 0.1	74.470	43.788	84.8
	4.95	0.070840 ± 0.00010	35,047 ± 47	25.1 ± 0.11	107.8 ± 0.1	72.470	42.612	87.9
holo	0.65	0.012240 ± 0.00011	44,921 ± 401	28.1 ± 0.38	85.9 ± 0.9	83.410	49.045	75.4
	1.0	0.018245 ± 0.00009	43,523 ± 224	32.9 ± 0.39	89.5 ± 0.5	70.910	41.695	94.1
	2.87	0.060289 ± 0.00018	50,112 ± 154	32.4 ± 0.15	72.7 ± 0.3	91.160	53.602	63.9
	5.0	0.109217 ± 0.00037	52,108 ± 176	31.6 ± 0.13	67.6 ± 0.3	95.060	55.895	58.0

[a:] The molecular mass *M* of the scattering particle was calculated according to $M = \frac{I(0)N_A}{c(\Delta\rho_M)^2}$ [25] with *I*(0) on the absolute scale (cm^{-1}), N_A the Avogadro's number, c the concentration of the scattering particle (g ml^{-1}) and $\Delta\rho_M$ the scattering mass contrast, which can be calculated as $\Delta\bar{\rho}\bar{v}$, where $\Delta\bar{\rho}$ is the average scattering-length density difference between the particle and its solvent (cm^{-2}) and $\Delta\bar{v}$ is its partial specific volume (cm^3 g^{-1}). $\Delta\bar{\rho}$ and $\Delta\bar{v}$ were calculated from the chemical composition of the solvent and solute using the *MULCh* webserver (http://smb-research.smb.usyd.edu.au/NCVWeb) [15]. [b:] The molecular mass of the scattering particle *M* was calculated from the Porod volume by multiplication with the reported protein density of 0.588 g ml^{-1} [13]. [c:] The fraction of the monomer (φ) was calculated according to $M = \varphi M_{theoMonomer} + (1-\varphi) M_{theoDimer}$, with $M_{theoMonomer}$ and $M_{theoDimer}$ representing the theoretical molecular mass of the *Te*LPOR apo- and holoprotein monomer and dimer, respectively. Apoprotein: $M_{theoMonomer}$ = 38,014 Da and $M_{theoDimer}$ = 76,028 Da; Holoprotein: $M_{theoMonomer}$ = 39,370 Da and $M_{theoDimer}$ = 78,740 Da; The theoretical molecular mass of the holoprotein monomer and dimer assumes a 1:1 load of the protein with Pchlide ($M_{Pchlide}$ = 612 Da) and NADPH (M_{NADPH} =744 Da). For clarity φ is expressed as a percentage. [*:] dataset merged for further analyses

Supplementary Table 3: *Te*LPOR ternary complex dimer modelling – best ClusPro models. *Te*LPOR dimer models were generated by the ClusPro docking server in homo-multimer mode as described in the Supplementary Methods section using the C-terminally extended *Te*LPOR model as monomer receptor. All models were compared against the experimental SAXS scattering curve for the holoprotein sample at high concentration (5.0 mg ml^{-1}) using the program OLIGOMER [8]. Form factor files for the monomer and the corresponding dimer were generated from the respective pdb file using FFMAKER. The generated models varied with respect to the favored interaction either being balanced, electrostatic, hydrophobic or a combination of Van-der-Waals and electrostatic interactions [26].

**Model type**	**Model**	**Representative image**	χ	R_g **(nm)**	**Monomer (%)**	**Dimer (%)**	**Distance (Å)**<br>**K197-NZ … *K197-NZ'***<br>**(K197-CA … *K197-CA'*)**
balanced	3b	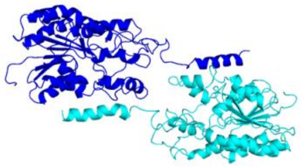	1.69	35.05	33	67	47.2<br>(53.6)
balanced	4b	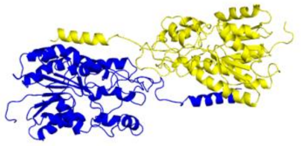	1.64	33.30	29	71	26.5<br>(36.8)
balanced	18b	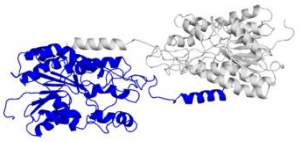	1.81	35.70	29	71	34.5<br>(44.5)
electrostatic favored	11e	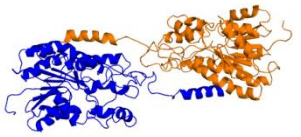	1.70	34.91	29	71	32.1<br>(49.8)
electrostatic favored	12e	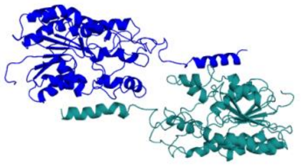	1.69	35.05	33	67	47.2<br>(62.8)
electrostatic favored	17e	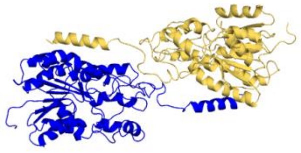	1.63	33.26	32	68	26.8<br>(43.3)
hydrophobic favored	1h	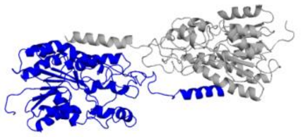	1.73	35.19	29	71	32.9<br>(50.5)
VdW+Elec	8Ve	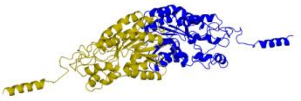	1.78	33.42	34	66	74.4<br>(59.2)
VdW+Elec	9Ve	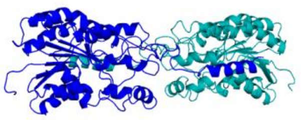	1.80	35.74	34	66	46.0<br>(58.7)
VdW+Elec	13Ve	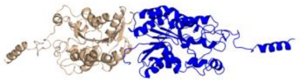	1.54	34.62	32	68	79.8<br>(64.0)

Supplementary Table 4: *Te*LPOR ternary complex dimer MD simulation – comparison of selected models. All models were compared against the experimental SAXS scattering curve for the holoprotein sample at high concentration (5.0 mg ml^{-1}) using the program OLIGOMER [8]. Form factor files for the monomer and the corresponding dimer were generated from the respective pdb file using FFMAKER.

**Model**	χ	R_g **(nm)**	**Monomer (%)**	**Dimer (%)**
best dimer 4b	1.64	33.30	29	71
MD dimer 25	1.53	33.71	54	46
MD dimer 489	1.67	33.04	59	41

Supplementary Table 5: Oligonucleotides used in this study

Oligonucleotide name	Oligonucleotide sequence 5´-3´[a]
*Te*LPOR_A302Stopp_for	CCAAGAACTATCGGCAGAGTAAAGTGATGAGCAAAAAGCC
*Te*LPOR_A302Stopp_rev	GGCTTTTTGCTCATCACTTTACTCTGCCGATAGTTCTTGG
*Te*LPOR_V279Stopp_for	TTTCGCCAGTCGGGGTAACACTGGAGCTGGGGT
*Te*LPOR_V279Stopp_rev	ACCCCAGCTCCAGTGTTACCCCGACTGGCGAAA
*Te*LPOR_P272Stopp_for	CGATGGTGGTCGCAGACTAAGAGTTTCGCCAGTCGGG
*Te*LPOR_P272Stopp_rev	CCCGACTGGCGAAACTCTTAGTCTGCGACCACCATCG

[a]: the introduced stop codon is highlighted in red

Supplementary Discussion

1.1 Protein purification and SAXS/AUC sample preparation.

The heterologously produced *Te*LPOR apoprotein was purified by immobilized metal ion affinity chromatography (IMAC) (Supplementary Fig. 1a) and preparative size-exclusion chromatography (SEC) (Supplementary Fig. 1b). Purification progress as well as the final protein samples were analyzed by SDS-PAGE (Supplementary Fig. 1c). As shown in Supplementary Fig. 1c the *Te*LPOR apoprotein could by purified to homogeneity with an estimated sample purity exceeding 95 %. After purification, the *Te*LPOR/NADPH/Pchlide ternary holoprotein complex was reconstituted by incubating equimolar amounts of protein with NADPH and Pchlide (see Materials and Methods). All steps were carried out in the dark to avoid substrate turnover. Light-dependent activity of the *Te*LPOR holoprotein SAXS sample is demonstrated in the main manuscript (Figure 1b). In contrast to the previously reported LPOR assay conditions [27], those samples lacked the additives dithiothreitol (DTT) and the detergent Triton X-100. This adjustment was made to avoid potential problems arising from detergent micelle formation, which might potentially impact our SAXS measurement. While the data presented in the main manuscript clearly shows light-dependent Pchlide turnover (Figure 1b), we also carried out a control experiment in which we supplied 70 µM DTT and 0.03 % (v/v) Triton X-100 to the holoprotein sample in reaction buffer and subsequently illuminated the sample. The corresponding light-dark difference spectra are shown in Supplementary Fig. 2a. As also shown in Figure 1b of the main manuscript, illumination of the sample results in a decrease of a band at around 640 nm (Pchlide substrate)[28] and a concomitant increase of an absorption band at around 675 nm (Chlide reaction product)[27,29], clearly demonstrating, that light-dependent Pchlide turnover proceeds both in the presence and absence of DTT and Triton. Taken together, while the reaction appears to proceed less efficient without DTT and Triton X-100 (Supplementary Fig. 2b), we nevertheless observed functionality of the protein under the conditions later employed in the here presented SAXS and AUC studies.

1.2 Multi-wavelength analytical ultra-centrifugation (AUC).

The data were analyzed with the standard c(s) model in SEDFIT with the frictional ratio as a floating parameter. As necessary parameters for this direct sedimentation boundary modelling, buffer density and viscosity were calculated incrementally using Sednterp 2.0

according to the given composition (1.0772 g/mL and 0.020277 P, respectively). Likewise, the partial specific volume was calculated incrementally according to the amino acid composition of the His_6-tagged *Te*LPOR (0.740 ml/g). As an approximation, preferential hydration and solvation effects in concentrated glycerol solutions (on apo- and holoprotein) as well as of NADPH/Pchlide binding on holoprotein were neglected by using an identical partial specific volume for all c(s) data fits. This assumption is valid, since the resulting c(s) distributions were only marginally altered when using conceivable alternative values for the partial specific volume volume (0.72 and 0.76 ml/g). A weight-averaged frictional ratio reflecting all sedimenting species according to their abundances is assigned by the c(s) algorithm to model the diffusion-corrected propagation of sedimentation boundaries.

The fitted sedimentation profiles for both apoprotein and holoprotein reflect the raw data sufficiently well, though the data fit is affected by the formation of a dynamic density and viscosity gradient due to redistribution of glycerol in the reaction buffer during the experiment (Supplementary Fig. 3a,b). A correction of this inhomogeneous solvent effect is possible for c(s) data evaluation using SEDFIT, but the necessary assumptions may not be entirely valid for the complex apoprotein/holoprotein system in reaction buffer [30]. Notwithstanding, the applied parametrization used in the present study resulted in robust data fits, e.g. the apparent molar mass of the predominant monomeric protein matches well with the calculated mass, thus corroborating a sufficient precision of the oligomer distributions found for *Te*LPOR and holoprotein.

A deconvolution into component sedimentation profiles using the extinction coefficients from absorption spectra of the three individual components (apoprotein, NADPH and Pchlide as depicted in Supplementary Fig. 3d,e and Figure 1e of the main manuscript) in reaction buffer led to distorted component concentrations. This finding indicates that, as mentioned in the main manuscript, various species exhibit chromatic shifts upon complex formation with distinct spectral features not representing a linear combination of the individual components' spectra. Indeed, significant chromatic shifts between independently dissolved Pchlide and as component in the holoprotein sample are recognizable in Supplementary Fig. 3d,e and Figure 1e of the main manuscript).

The absorption spectrum of the holoprotein sample (Supplementary Fig. 3d,e and Figure 1e of the main manuscript) as well as the extracted absorption spectra of the sedimenting material (Supplementary Fig. 3f) corresponding to the peaks of monomeric protein (0.47-1.50 S) and dimer (1.50-2.20 S) allowed for a robust quantitative assessment of the oligomer distribution:

The absorption spectrum of a 0.5 mg/mL holoprotein sample (Supplementary Fig. 3d,e and Figure 1e of the main manuscript) resembles in the region dominated by Pchlide (> 400 nm) the sum of the individual absorption spectra of apoprotein, NADPH and Pchlide in a molar ratio of approximately 1/0.67/0.67. Assuming that the sum of all species' chromophoric properties at wavelengths larger than 400 nm is essentially identical to that of the three independently dissolved components' spectra, this suggests that approximately each a third of NADPH and Pchlide escaped detection by forming large aggregates/particulates removed by a prior sedimentation step or sedimentation to the bottom during the initial phase of the experiment.

Furthermore, each a third of Pchlide forms soluble aggregates along with NADPH which may also contain a few percent of apoprotein/holoprotein, as deduced from the respective absorption spectra, featuring a nearly identical absorption at 438 nm for both monomer and dimer combined as well as the soluble Pchlide/NADPH aggregates (Supplementary Fig. 3f).

In conclusion, approximately 67 % of total protein represents apoprotein, predominantly in the monomeric state, whereas 33 % co-sediments with NADPH and Pchlide indicating holoprotein formation. The ratio of holoprotein monomer to dimer is approximately 2:1 as indicated by the ratio of the monomer/dimer absorption at 438 nm. This molar ratio is in good agreement with the relative monomer/dimer signal amplitudes from the c(s) distributions at 340 and 437 nm (Supplementary Fig. 3c and Figure 1c,d of the main manuscript). Overall, holoprotein monomers and dimers represent approximately 22 % and 11 % of total protein, respectively, resulting in an overall monomer to dimer ratio of 89 % to 11 %.

To test whether both the monomeric and the dimeric holo-protein species are show light-dependent activity, we performed an MWA-AUC experiment, where we analysed the same holoprotein sample (containing a mixture of monomeric and dimeric holoprotein) after illumination (Supplementary Fig. 3, g and h). As deduced from both the c(s) distributions (Supplementary Fig. 3g) as well as from the extracted absorbance spectra (Supplementary Fig. 3h) the ratio of holoprotein monomer:dimer loaded with Pchlide/Chlide increases after illumination from approximately 2:1 to 3-4.5:1. This suggest that the stability of holoprotein dimers is lower than that of monomers under the applied conditions, e.g. resulting from a higher rate of Pchlide/Chlide loss for dimers and/or dissociation of holoprotein dimers into monomers. This observation independently supports our model presented in Figure 5 of the main manuscript, corroborating previous studies on LPORs [31,32], thus providing a rational for light-dependent disintegration of prolamellar bodies. In our setup, unfortunately, we only

observed minor light-dependent Chlide production as discernible from the low, but nevertheless well detectable, 671 nm absorption band (Supplementary Fig. 3h), which is commonly associated with Chlide production [33]. Interestingly, the formed Chlide product co-sediments with the both the monomeric and the dimeric species, indicating that the formed Chlide is still protein bound. This, together with the spectral signature of the product band (λ_{max} = 671 nm), suggests that the product-containing monomeric and dimeric species represent the Chlide/*Te*LPOR binary complexes [33]. In conclusion, these results suggest that either both the holoprotein monomers as well as the holoprotein dimers show light-dependent activity, or that the monomeric Chlide/*Te*LPOR binary complex, detected by MWA-AUC, results from the dissociation of the product-containing dimer. Given the altered monomer:dimer distribution which we observed for the illuminated holoprotein sample (monomer:dimer 3-4.5:1; Supplementary Fig. 3g) as compared to the dark-reconstituted holoprotein (monomer:dimer 2:1; Figure 1d, Supplementary Fig. 3c), we would favor the latter interpretation. However, since we were not able to separate the monomeric and dimeric holoprotein species chromatographically, to independently test their activity, we are at present unable to clearly delineate between the two possibilities.

1.3 SAXS data evaluation and selection of representative data sets.

The concentration normalized SAXS data for all samples is shown in Supplementary Fig. 4. The Guinier plots of the corresponding SAXS data at low q values (Supplementary Fig. 5) suggest that all apoprotein samples are essentially free of aggregates (Supplementary Fig. 5a-c), while the holoprotein sample appears to be minimally aggregated (Supplementary Fig. 5d). Given the AUC-based observation that the formation of the ternary complex results in an increase in dimerization of *Te*LPOR, we analyzed our data with regard to a concentration dependence of the radius of gyration R_g, the forward scattering intensity ($I(0)$) and the Porod volume (V_p) (Supplementary Fig. 6), and calculated the molecular mass M of the corresponding scattering particles for each sample of the respective concentration series (Supplementary Table 2, Supplementary Fig. 6e,f). For all apoprotein samples there is, at best, very weak concentration dependence, with the SAXS-derived molecular masses closely matching the theoretically-expected M of the *Te*LPOR monomer. Assuming the presence of a minor dimeric component, we calculated the fraction of the corresponding monomer for each sample of the concentration series (Supplementary Table 2). Here a certain sample-to-sample variability is observed. In this regard, the apoprotein sample 2 (apo 2) shows the lowest

variability, with a monomer content between 97% and 100%, when considering *M* from *I*(0), and between 81% and 99%, when considering *M* from V_p. We therefore decided to use for all further analyses the SAXS data of the apo 2 sample and merged a low and high concentration dataset to improve data quality (see Supplementary Table 1 and 2). In contrast to the apoprotein samples, we observe a more pronounced concentration dependence of the SAXS derived *M* for the holoprotein. Moreover, compared to the apoprotein data, the SAXS-derived *M*, *I*(0) and R_g values are clearly larger for the holoprotein samples. Hereby, the SAXS derived *M* exceeds the theoretical *M* of the corresponding *Te*LPOR monomer, hinting at an increased dimer content. This corroborates the above presented AUC data and suggests that the formation of the *Te*LPOR/NADPH/Pchlide ternary complex results in dimerization of *Te*LPOR. For the highest concentrated holoprotein sample we estimate a dimer content between 32 % (*M* from *I*(0)) and 42 % (*M* from V_p). For the lowest concentrated sample a dimer content between 14 % (*M* from *I*(0)) and 25 % (*M* from V_p) is obtained. Therefore, for further, more detailed, analyses, we directly used the SAXS data of the highest concentrated sample.

1.4 Homology modelling, C-terminal extension and apoprotein monomer model evaluation.

1.4.1 Evaluation of alternative C-terminally extended TeLPOR models

The homology model of the LPOR enzyme of *Thermosynechococcus elongatus* (*Te*LPOR) was constructed using YASARA Structure Version 16.6.24 [19,20] based on the *E. coli* 7a-hydroxysteroid dehydrogenase (PDB: 1AHI) [4] employing the previously published homology model of the LPOR of *Synechocystis* sp. (*Ss*LPOR) [5] as template. This model, to which we refer to as *Te*LPOR core domain, represents the monomer subunit of the enzyme as ternary (holoprotein) complex with bound NADPH and Pchlide [5]. The corresponding apoprotein monomer was generated by removing NADPH and Pchlide from the model. Compared to the construct studied by SAXS, this model lacks 20 N-terminal residues constituting the His_6-tag, and is missing 31 amino acids at the C-terminus, which were not covered by the template used for homology-model building. Previous secondary structure predictions as well as circular dichroism (CD) spectroscopic data for the homologous wheat LPOR (42% identical amino acids to *Te*LPOR), solubilized from the prolamellar-body fraction of wheat, suggested that the missing C-terminal region should possess α-helical structure [34]. Our own secondary structure predictions for *Te*LPOR, carried out using the NPS@ consensus secondary structure prediction webserver (https://npsa-prabi.ibcp.fr/cgi-

bin/npsa_automat.pl?page=/NPSA/npsa_seccons.html) [1], confirm those earlier predictions (Supplementary Fig. 7a). Additionally, *Te*LPOR homology models were built using Phyre^2 [2] (http://www.sbg.bio.ic.ac.uk/phyre2/html/page.cgi?id=index) and I-TASSER [35](https://zhanglab.ccmb.med.umich.edu/I-TASSER/). All obtained models predict α-helical secondary structure for the C-terminal extension, either as one continuous α-helix (Phyre$_2$, Supplementary Fig. 7b) or establishing a (coil)helix-turn-helix motif (Phyre^2, I-TASSER; Supplementary Fig. 8b). Using this information, the originally built *Te*LPOR core domain model was manually extended C-terminally by adding protruding α-helical elements possessing different conformation, i.e. as observed in the different extended homology models (Figure 3a, main manuscript, Supplementary Fig. 10a). The first model (*Te*LPOR-C$_{helix}$, shown in red in Figure 3a) was built manually with YASARA with the C-terminal protruding α-helix optimally fitting into the DAMMIN/DAMMIF/GASBORP-generated envelopes. Two alternative models (*Te*LPOR-C$_{loop1helix}$, shown in blue in Figure 3a, Supplementary Fig. 10a; *Te*LPOR-C$_{loop2helix}$, shown in orange in Figure 3a, Supplementary Fig. 10a) were built by loop modelling using YASARA [36]. Using those models three 37.75 ns Molecular Dynamic (MD) simulations (without bound NADPH and Pchilde ligands) were performed and the resulting trajectories were analyzed with regard to core domain (residues 1-285) stability in terms of the backbone root mean square deviation (RMSD) (Supplementary Fig. 10b), conformational flexibility of the C-terminal extension (Figure 3d-f, main manuscript) and secondary structure (Supplementary Fig. 10c-e). In all simulations, the C-terminal extension samples multiple conformations (Figure 3d-f, main manuscript), while its overall secondary structure remains stable over the simulation time (Supplementary Fig. 10c-e; compare C-terminal α-helix). Likewise, in all three simulations, no dramatic unfolding events or conformational changes are observed for the *Te*LPOR core domain (Supplementary Fig. 10c-e). This suggests that even in the absence of the Pchlide substrate and the NADPH cofactor the *Te*LPOR structure remains stable, at least over the here employed simulation times. Out of the three extended *Te*LPOR homology models the *Te*LPOR-C$_{helix}$ model displays the lowest core domain RMSD (Supplementary Fig. 10b) and possesses the highest secondary structure stability (Supplementary Fig. 10c). Subsequently, all starting models, as well as 150 250-ns snapshots of the corresponding trajectories were independently cross validated against the experimental SAXS data by using CRYSOL (Figure 3g, main manuscript). All models were scored by comparing the CRYSOL-derived theoretical scattering curve of the respective model with the experimental SAXS data of the apoprotein sample (apo 2). To enable a direct comparison between different models no constant was subtracted in CRYSOL. Model validity was

assessed by comparing the χ values for the corresponding CRYSOL fit, with lower χ values as indicator for a better model. Initially the three different C-terminally extended homology models were compared to the *Te*LPOR core domain model (Figure 3a, main manuscript) lacking a C-terminal extension. The fit of the experimental scattering data of the apoprotein against the *Te*LPOR core domain model yielded a χ value of 2.864 (pink dashed line). Addition of any of the C-terminal extensions significantly improved the fit resulting in χ values of 1.654 (*Te*LPOR-C_{helix}, red solid line), 2.140 (*Te*LPOR-$C_{loop1helix}$, blue dashed line) and 1.848 (*Te*LPOR-$C_{loop2helix}$, orange dashed line) (Figure 3b,c, main manuscript). To account for conformational flexibility of the corresponding C-terminal extensions, we used CRYSOL to calculate a theoretical scattering curve for 150 250-ns snapshots from each MD trajectory of the C-terminally extended *Te*LPOR models, and fitted the resulting data against the experimental scattering curve of the apoprotein sample. To potentially identify conformations that better fit the experimental data, the obtained χ values were plotted against the simulation time (Figure 3g, main manuscript). For the *Te*LPOR-C_{helix} trajectory, the CRYSOL-derived χ values remained very similar over the whole simulation time ($\chi_{average}$ = 2.6 ± 0.1) (Figure 3g, main manuscript, solid red line), although a large displacement of the C-terminal helix occurred during the simulation (Figure 3d, main manuscript). This indicates that a freely moving C-terminal helix is accommodated by our SAXS data. In contrast, for *Te*LPOR-$C_{loop1helix}$ and *Te*LPOR-$C_{loop2helix}$ models the χ values significantly deviated from the starting value during the simulation, yielding average χ values of 3.8 ± 0.9 (*Te*LPOR-$C_{loop1helix}$, Figure 3g, blue dashed line) and 4.2 ± 0.8 (*Te*LPOR-$C_{loop2helix}$, Figure 3g, orange dashed line). For all three models, the conformers sampled during the corresponding trajectories yielded a worse fit than the initially constructed starting model.

1.4.2 Evaluation of alternative core domain and full-length TeLPOR models

To generate and evaluate alternative *Te*LPOR core domain models, i.e. deviating from the overall core domain fold of the *Ss*LPOR homology model built by Townley *et al.* [5], we used I-TASSER to automatically generate homology models of the full-length *Te*LPOR enzyme, which included the putative C-terminal helical extension. I-TASSER generated five very similar homology models in which the C-terminal extension possesses α-helical secondary structure, which however, in contrast to the above described models, packs against the core domain Rossmann-fold (Supplementary Fig. 8a,b). All models (Supplementary Fig. 8b) were scored against the experimental SAXS data by fitting the experimental data against the CRYSOL-calculated scattering curve of each model (Supplementary Fig. 8c,d; dashed lines, shades of green). Compared to the above described *Te*LPOR core domain model

(Supplementary Fig. 8c, χ = 2.864; pink dashed line), and the model with protruding α-helical elements (Supplementary Fig. 8c, +C_{helix}; solid red line), all I-TASSER generated models yielded significantly worse χ values between 3.382 (model 5) and 4.103 (model 3) (Supplementary Fig. 8c).

1.4.3 Evaluation of alternative TeLPOR models containing unordered N- and C-terminal extensions

As outlined above, compared to the construct measured by SAXS, the *Te*LPOR homology model lacks 31 C-terminal residues, as well as 20 N-terminal amino acids that constitute the His_6-tag. To account for the possibility that both structural elements adopt a flexible, unstructured conformation, we modelled the missing elements as flexible ensemble with EOM (Supplementary Fig. 9) and compared the obtained ensemble scattering curves to the CRYSOL-derived theoretical scattering curves of the *Te*LPOR core domain model (Supplementary Fig. 9, *Te*LPOR core, dashed pink line) and the best C-terminally extended model in which the missing C-terminal residues are modelled as protruding α-helix (Supplementary Fig. 9, +C_{helix}, solid red line). Three ensembles were considered, i) for which the N-terminal His_6-tag was considered flexible (Supplementary Fig. 9,+N_{flex}, dashed blue line), with the model otherwise lacking the C-terminal extension, ii) the C-terminally extended model completed by modelling the N-terminal His_6-tag as flexible ensemble (Supplementary Fig. 9, +C_{helix}+N_{flex}, dashed green line), and iii) the *Te*LPOR core domain model completed N- and C-terminally by modelling all missing residues as flexible ensemble (Supplementary Fig. 9, +C_{flex}+N_{flex}, dashed orange line). Compared to the *Te*LPOR-C_{helix} model (χ = 1.654), consideration of a flexible N-terminal His_6-tag did not improve the fit (+N_{flex}+C_{helix}: χ = 2.907). Likewise, a model possessing only a flexible C-terminal His_6-tag (+N_{flex}: χ = 2.771), as well as a model for which both the missing N-terminal and the missing C-terminal residues were modelled as flexible ensemble (Nflex+Cflex: χ = 3.010) resulted in worse χ values.

1.4.4. Evaluation of alternative TeLPOR models possessing a flexible insertion loop element.

To address the possibility that structural elements other than the immediate termini adopt a flexible/disordered conformation, thereby accounting for our SAXS data, we performed additional modeling studies with EOM, in which certain parts of the *Te*LPOR structure are modeled as flexible/disordered ensemble. The resulting theoretical ensemble scattering curves were then, as before, compared against the experimental SAXS data. From literature, it is

known that LPORs possess a 25-30 residue long sequence stretch, which is not present in related short-chain dehydrogenases [5]. In *Te*LPOR this corresponds to the residues 154 to 185. In our *Te*LPOR homology model, in analogy to the *Ss*LPOR model of Townley and coworkers [5], a part of this insertion loop is modeled as α-helix. This is in excellent agreement with recent, unpublished NMR data provided in the doctoral thesis of David Robert Armstrong (2014, University of Sheffield, UK)[6], where TALOS-N based secondary structure predictions suggested α-helical secondary structure for the residues around residue 170. To theoretically assess if this insertion loop might be disordered, we performed disorder predictions for *Te*LPOR using the MetaDisorder webserver, which combines multiple disorder prediction algorithms [3]. Here, potentially disordered regions were only identified at the immediate N-terminus, for the C-terminal extension (residue 285 onwards) and, albeit with weak disorder tendency, also for the potential LPOR insertion loop (residues 160 to 185) (Supplementary Fig. 9c). Supplementary Fig. 9d depicts a multiple sequence alignment of *Te*LPOR and a related 7α-hydroxysteroid dehydrogenase from *E. coli* (EcHSD, PDB-ID: 1AHI, [4]), illustrating the position of the insertion loop. Subsequently, we evaluated different models with EOM (Supplementary Fig. 9g,h), which differed only with respect to the regions that were modeled as disordered/flexible ensemble: In model 1 the complete insertion loop (residues 154-185; as defined by Townley and coworkers [5] (Supplementary Fig. 9e, highlighted in red) and the C-terminal extension were considered as disordered/flexible (Supplementary Fig. 9e, highlighted in blue), while in model 2, the C-terminal extension was kept rigid as in our best C-terminally extended model, whereas the insertion loop was modeled as disordered ensemble (Supplementary Fig. 9e). In model 3 only a part of the insertion loop (residues 150-160) (Supplementary Fig. 9f, highlighted in red), as well as the complete C-terminal extension (Supplementary Fig. 9d, highlighted in blue) were assumed to be disordered. In model 4 the same part of the insertion loop as in model 3 (Supplementary Fig. 9f, highlighted in red) was assumed disordered, while the C-terminal extension was kept rigid. The latter two scenarios are based on NMR data presented as part of the doctoral thesis of David Robert Armstrong (2014, University of Sheffield, UK)[6], which by NMR experiments showed that the residues between 150 and 160 show increased mobility, while the rest of the insertion loop likely adopts α-helical structure. All four models were scored against our experimental SAXS data (Supplementary Fig. 9g,h) yielding χ values between χ = 2.124 (model 1) and χ = 2.620 (model 4), which are significantly worse as compared to our best initial model in which both the C-terminal extension as well as the insertion loop were kept rigid (χ = 1.654; see Supplementary Fig. 9g,h; +C_{helix} model shown for comparison). In

conclusion, the C-terminally extended model in which the missing C-terminal 31 amino acids are modelled as protruding α-helix (*Te*LPOR-C_{helix}), best fits the experimental SAXS data for the apoprotein, and is thus presented as final (best) *Te*LPOR apoprotein model in the main manuscript.

1.4.5 The C-terminally extended TeLPOR model fits the SAXS-derived envelopes of the monomeric apoprotein.

For cross validation, the above described C-terminally extended *Te*LPOR homology model (Figure 3j, main manuscript red cartoon representation) as well as the *Te*LPOR core domain model (Figure 3j, blue cartoon representation) were superimposed to the averaged and filtered SITUS-derived *ab initio* envelopes obtained by DAMMIF, DAMMIN and GASBORP. As also outlined in the main manuscript, all three programs yielded very similar *ab initio* models, with a bowling-pin like shape, consisting of a larger and smaller subdomain. The *Te*LPOR Rossmann-fold core domain is hereby nicely accommodated by the larger subdomain envelope, whereas the C-terminal α-helical extension could account for the smaller subdomain (Supplementary Fig. 11 and Figure 3j of the main manuscript).

1.5 Modelling and validation of the NADPH/Pchlide/TeLPOR ternary complex dimer.

1.5.1 Protein-protein docking suggests a physically feasible mode of dimerization.

The dimeric structure of the NADPH/Pchlide/TeLPOR ternary complex (holoprotein) was modelled by homo-multimer protein-protein docking employing the ClusPro webserver [26,37-40]. The ClusPro server (https://cluspro.org) is a widely used protein-protein docking tool, that enables the direct docking of interacting proteins by utilizing three computational steps: i) rigid-body docking, sampling billions of conformations, ii) root-mean-square deviation (RMSD)-based clustering of the 1000 lowest-energy structures of the initial pool to identify the largest clusters that represent the most likely model of the complex and iii) refinement of selected structures by energy minimization. The server generates four sets of models, scored according to the type of interaction that mainly drives complex formation. Scoring is achieved by utilizing different energy functions to derive complex models with i) balanced interactions, ii) favorable electrostatic interactions, iii) favorable hydrophobic interactions and iv) models for which mainly van der Waals and electrostatic interactions account for complex formation [26]. Two types of monomer models were used as input for ClusPro docking. First, the *Te*LPOR core domain model lacking 31 residues at the C-terminus, and secondly, a C-terminal

extended model in which the missing 31 C-terminal residues were modeled as protruding α-helix (see Supplementary Discussion section 1.4.1) No distance constrains or attractive/repulsive interactions were utilized to steer the clustering process. Overall, 78 *Te*LPOR core domain dimer models and 81 C-terminally extended dimer models were generated by ClusPro. Model validity was scored by comparing the theoretical scattering curve of the corresponding monomer/dimer mixture (obtained by OLIGOMER [8]) against the corresponding SAXS data of the holoprotein (5.0 mg ml^{-1} dataset). The program OLIGOMER hereby fits the experimental scattering curve from a multicomponent mixture of proteins (here monomer and dimer). The program requires a set of form factors for each component present in the mixture. Form-factor files of the monomer and dimer model were generated using FFMAKER. Apart from a χ value for the fit against the experimental data, OLIGOMER provides an estimate of the R_g and the computed monomer and dimer fractions. The complete model evaluation effort is summarized in Supplementary Fig. 12 and 13. In all cases the C-terminally extended dimer models yield better fitting models in terms of χ and R_g. The properties of 10 the best fitting C-terminally extended *Te*LPOR dimer models are given in Supplementary Table 3 (models and χ values also shown in Figure 4a of the main manuscript).

The final best model was selected based on the χ value for the fit of the theoretical scattering curve of the oligomeric mixture and the experimental data. Additionally, experimental cross-linker based distance constrains, identified for the homologous POR A protein of *Arabidopsis thaliana* (*At*PORA)(42% identical amino acid positions), were considered. Here it was shown by cross-linking studies using the BS3 (bis(sulfosuccinimidyl)suberate) crosslinker, which covalently links lysine residues via the NZ atoms, that the 281/284 lysine residue of two *At*PORA subunits (corresponding to K194 and K197 of *Te*LPOR) come closer to each other upon formation of NADPH/Pchlide/*At*PORA ternary complex [41]. Based on the comparison of the dynamic behavior of 766 molecular dynamics simulations (represented in the Dynameomics database, www.dynameomics.org) Merkley *et al.*[42] recently concluded that for the BS3 cross-linker a distance constraint of 26-30 Å between the CA atoms of two lysine is appropriate to enable cross linking. Given this limit, we selected the *Te*LPOR dimer model, which shows the lowest K197-CA … *K197-CA'* distance, and yielded the best χ value for the fit between the theoretical scattering curve of the oligomeric mixture and the experimental data. This analysis identifies model 4b (in the main manuscript designated as best dimer 4b) as the best model with a χ value of 1.64 and a K197-CA … *K197-CA'* distance of 36.8 Å (Supplementary Table 3). This model was used as the representative model of the dimeric

NADPH/Pchlide/*Te*LPOR ternary complex. Please note that 8 out of the 10 best dimer models show a very similar mode of dimerization mediated by the active-site surface patch and the protruding C-terminal α-helix.

1.5.2 The NADPH/Pchlide/TeLPOR ternary complex dimer is stable during MD simulation.

To address if the obtained NADPH/Pchlide/*Te*LPOR ternary complex dimer model is stable, we performed an MD simulation of the above described dimer 4b. The simulation was set up in identical fashion as described for the apoprotein models, and was run until convergence of the RMSD (Supplementary Fig. 14a). Snapshots were extracted from the trajectory every 100 ps. The resulting pdb coordinates were scored by comparing the theoretical scattering curve of the corresponding monomer/dimer mixture (obtained by OLIGOMER [8]) against the corresponding SAXS data of the holoprotein (5.0 mg ml^{-1} dataset) (Supplementary Fig. 14b). During the simulation, the χ value initially decreases from about 1.64 to 1.53 within the first 2.5 ns, after which a steady increase is observed finally converging after about 20 ns to reach a constant level of approx. χ = 1.7. This coincides with convergence of the RMSD (compare Supplementary Fig. 14a and b). During the simulation, the OLIGOMER-derived apparent radius of gyration (R_g) decreases marginally, which coincides with a reduction of the OLIGOMER-derived apparent dimer content from about 70 % (initial structure) to about 40 % at the end of the simulation (Supplementary Fig. 14c). Please note that the latter value is in excellent agreement with the dimer content estimated from the Porod volume (Supplementary Table 2 and Table 1 of the main manuscript). Over the simulation time, a slight rearrangement of the dimer interface, i.e. manifested in a minor displacement of the C-terminal helix, is observed (Supplementary Fig. 14d). Based on this analysis, an early snapshot (snapshot 25), showing the best χ value for the fit against the experimental SAXS data (χ = 1.53) and a late snapshot (snapshot 489), representing the dimer structure after convergence of the RMSD, were selected for further analyses. The direct superimposition of those snapshots to the initial dimer 4b structure is shown in Supplementary Fig. 14d. Supplementary Fig. 15 depicts the direct comparison of the theoretical scattering curves for the corresponding monomer/dimer mixtures as inferred by OLIGOMER. All dimer models more or less equally well fit to the experimental data (χ values between 1.53 and 1.67), more pronounced differences were found for the OLIGOMER-derived apparent monomer/dimer ratio (Supplementary Table 4). While for the initial dimer model 4b a dimer content of about 70% was derived by OLIGOMER, for both MD-derived snapshots lower dimer content was inferred. In particular, for the late snapshot (MD dimer 489) a dimer content of about 40% is obtained, which is in excellent

agreement with the dimer content estimated from the Porod volume (42% dimer; see Supplementary Table 2 and Table 1 of the main manuscript). Since the mode of interaction, as well as the overall dimer structure did not change dramatically during the simulation, only the data for the initial ClusPro-derived dimer 4b is shown in the main manuscript.

1.5.3 Dimerization is driven by both electrostatic and hydrophobic interactions mediated by conserved residues on both the Rossmann-fold core domain and the C-terminal helix

The flexibility of the overall dimer structure as well as a potential rearrangement of the dimer interface during the MD simulation was analyzed by calculating the per residue root mean square fluctuation (RMSF) for the backbone atoms on a 250 ps time window over the simulation trajectory (Figure 5a, red solid line, main manuscript). For the initial dimer 4b structure the inter-chain contacts were determined from the pdb coordinates using the WHAT-IF web service (https://swift.cmbi.umcn.nl). Residues involved in dimerization are highlighted as grey vertical lines in Figure 5a. Increased RMSF values were only observed for interface residues centered around reside 100, for the residues centered on residue 155 and several residues on the C-terminal helix. However, when we compare the per residue RMSF values obtained from the simulation of the ternary complex dimer, to the corresponding values of the corresponding apoprotein monomer simulation (Figure 5a, blue solid line) it is directly apparent, that free movement of the C-terminal helix (Figure 3d, main manuscript) is hindered by dimerization, as much higher RMSF values are observed for this structural element in the monomer simulation (Figure 5a, compare red and blue solid lines).

In terms of charge and hydrophobicity, the C-terminal helix possesses amphipathic character (Supplementary Fig. 15a), with the hydrophobic portion, mainly constituted by L311, L314 and L318, packing against the opposite subunit (Supplementary Fig. 16b). To analyze the conservation of the dimer interface, we generated a sequence logo for LPORs based on 315 LPOR amino acid sequences from plants and bacteria (Figure 4b, main manuscript). Interestingly, most of the residues of the dimer interface are highly conserved within the LPOR family (Figure 4b; main manuscript, highlighted by orange asterisk). Additionally, the surface hydrophobicity of the two monomers of the *Te*LPOR dimer, was analyzed by employing the hpatch tool of the Rosetta protein design software [9,10], which identifies and scores clusters of hydrophobic atoms referred to as hydrophobic patches [43] (Supplementary Fig. 17). In the initial ClusPro-derived dimer 4b, as well as in the two MD-derived dimer models (MD dimer 25 and 489), the C-terminal helix covers a hydrophobic surface patch on the Rossmann-fold core domain, mostly formed by evolutionary conserved (Figure 4b, main

manuscript) residues in the region between residue 102-117 and 194-208. The corresponding hydrophobic surface at the C-terminus is largely constituted by conserved residues of the C-terminal helix (L311, L314 and L318) (Supplementary Fig. 16b, Figure 4b, main manuscript).

To better understand the nature of the dimer interaction, using the Adaptive Poisson Boltzmann Solver [11] plugin for Pymol, we calculated the electrostatic surface potential, of the two monomers of the *Te*LPOR dimer and superimposed those onto the dimer structure. This allowed us to individually visualize the charge distribution along the dimer interface (Supplementary Fig. 18). Interface residues were identified using the WHAT-IF web service. Interface residues are highlighted according to type on chain A of the dimer, while for chain B the electrostatic potential distribution mapped onto the protein surface is shown (Supplementary Fig. 18). In the initial ClusPro-derived dimer 4b, as well as in the two MD-derived dimer models (MD dimer 25 and 489), a limited number of charged residues on both the C-terminal helical extension, as well as on the Rossmann-fold core domain, contribute to the dimerization process (highlighted as red and blue spheres on subunit 1; Supplementary Fig. 18). The majority of the interfacial residues though are hydrophobic (shown as white spheres in Supplementary Fig. 18). During the MD simulation of the dimer, the dimer interface only changes minimally, retaining the overall interaction found in the ClusPro-derived starting structure. (Supplementary Fig. 16b; Supplementary Fig. 17 and 18).

In conclusion, while during the MD simulation some minor rearrangements of the dimer interface take place, the overall mode of dimerization, driven by hydrophobic and electrostatic interactions remains unchanged, indicative of a stable dimer interface. The dimer interaction seems largely driven by hydrophobic interactions, while the interface appears interspersed with a number of charged and polar residues. Moreover, residues involved in the interaction, both on the Rossmann-fold core domain as well as on the C-terminal helix, are conserved within the LPOR enzyme family. Based on this observation it is tempting to speculate that the observed dimer interface, and hence likely also the suggested mode of dimerization, is evolutionary conserved in LPORs.

Supplementary References

1 Combet, C., Blanchet, C., Geourjon, C. & Deleage, G. NPS@: Network Protein Sequence Analysis. *Trends in biochemical sciences* **25**, 147-150, doi:Doi 10.1016/S0968-0004(99)01540-6 (2000).

2 Kelley, L. A., Mezulis, S., Yates, C. M., Wass, M. N. & Sternberg, M. J. The Phyre2 web portal for protein modeling, prediction and analysis. *Nature protocols* **10**, 845-858, doi:10.1038/nprot.2015.053 (2015).

3 Kozlowski, L. P. & Bujnicki, J. M. MetaDisorder: a meta-server for the prediction of intrinsic disorder in proteins. *BMC bioinformatics* **13**, 111, doi:10.1186/1471-2105-13-111 (2012).

4 Tanaka, N. *et al.* Crystal structures of the binary and ternary complexes of 7 alpha-hydroxysteroid dehydrogenase from Escherichia coli. *Biochemistry* **35**, 7715-7730, doi:Doi 10.1021/Bi951904d (1996).

5 Townley, H. E., Sessions, R. B., Clarke, A. R., Dafforn, T. R. & Griffiths, W. T. Protochlorophyllide oxidoreductase: A homology model examined by site-directed mutagenesis. *Proteins* **44**, 329-335, doi:Doi 10.1002/Prot.1098 (2001).

6 Armstrong, D. R. *Structural and Functional Studies of the Light-Dependent Protochlorophyllide Oxidoreductase Enzyme*, The University oF Sheffield, (2014).

7 Wriggers, W. Conventions and workflows for using Situs. *Acta Crystallogr D* **68**, 344-351, doi:10.1107/S0907444911049791 (2012).

8 Konarev, P. V., Volkov, V. V., Sokolova, A. V., Koch, M. H. J. & Svergun, D. I. PRIMUS: a Windows PC-based system for small-angle scattering data analysis. *J Appl Crystallogr* **36**, 1277-1282, doi:10.1107/S0021889803012779 (2003).

9 Kuhlman, B. & Baker, D. Native protein sequences are close to optimal for their structures. *P Natl Acad Sci USA* **97**, 10383-10388, doi:DOI 10.1073/pnas.97.19.10383 (2000).

10 Rohl, C. A., Strauss, C. E. M., Misura, K. M. S. & Baker, D. Protein structure prediction using rosetta. *Numerical Computer Methods, Pt D* **383**, 66-+ (2004).

11 Baker, N. A., Sept, D., Joseph, S., Holst, M. J. & McCammon, J. A. Electrostatics of nanosystems: application to microtubules and the ribosome. *Proc Natl Acad Sci U S A* **98**, 10037-10041, doi:10.1073/pnas.181342398 (2001).

12 Pernot, P. *et al.* Upgraded ESRF BM29 beamline for SAXS on macromolecules in solution. *J Synchrotron Radiat* **20**, 660-664, doi:10.1107/S0909049513010431 (2013).

13 Petoukhov, M. V. *et al.* New developments in the ATSAS program package for small-angle scattering data analysis. *J Appl Crystallogr* **45**, 342-350, doi:10.1107/S0021889812007662 (2012).

14 Wilkins, M. R. *et al.* Protein identification and analysis tools in the ExPASy server. *Methods in molecular biology* **112**, 531-552 (1999).

15 Whitten, A. E., Cai, S. Z. & Trewhella, J. MULCh: modules for the analysis of small-angle neutron contrast variation data from biomolecular assemblies. *J Appl Crystallogr* **41**, 222-226, doi:10.1107/S0021889807055136 (2008).

16 Franke, D. & Svergun, D. I. DAMMIF, a program for rapid ab-initio shape determination in small-angle scattering. *J Appl Crystallogr* **42**, 342-346, doi:10.1107/S0021889809000338 (2009).

17 Svergun, D. I. Restoring low resolution structure of biological macromolecules from solution scattering using simulated annealing (vol 76, pg 2879, 1999). *Biophys J* **77**, 2896-2896 (1999).

18 Svergun, D. I., Petoukhov, M. V. & Koch, M. H. J. Determination of domain structure of proteins from X-ray solution scattering. *Biophys J* **80**, 2946-2953, doi:Doi 10.1016/S0006-3495(01)76260-1 (2001).

19 Krieger, E. & Vriend, G. YASARA View-molecular graphics for all devices-from smartphones to workstations. *Bioinformatics* **30**, 2981-2982, doi:10.1093/bioinformatics/btu426 (2014).

20 Krieger, E. & Vriend, G. New ways to boost molecular dynamics simulations. *Journal of computational chemistry* **36**, 996-1007, doi:10.1002/jcc.23899 (2015).

21 Svergun, D., Barberato, C. & Koch, M. H. J. CRYSOL - A program to evaluate x-ray solution scattering of biological macromolecules from atomic coordinates. *J Appl Crystallogr* **28**, 768-773, doi:Doi 10.1107/S0021889895007047 (1995).

22 Bernado, P., Mylonas, E., Petoukhov, M. V., Blackledge, M. & Svergun, D. I. Structural characterization of flexible proteins using small-angle X-ray scattering. *J Am Chem Soc* **129**, 5656-5664, doi:10.1021/ja069124n (2007).

23 Tria, G., Mertens, H. D. T., Kachala, M. & Svergun, D. I. Advanced ensemble modelling of flexible macromolecules using X-ray solution scattering. *Iucrj* **2**, 207-217, doi:10.1107/S205225251500202x (2015).

24 Humphrey, W., Dalke, A. & Schulten, K. VMD: visual molecular dynamics. *Journal of molecular graphics* **14**, 33-38, 27-38 (1996).

25 Trewhella, J. *et al.* 2017 publication guidelines for structural modelling of small-angle scattering data from biomolecules in solution: an update. *Acta crystallographica. Section D, Structural biology* **73**, 710-728, doi:10.1107/S2059798317011597 (2017).

26 Kozakov, D. *et al.* The ClusPro web server for protein-protein docking. *Nature protocols* **12**, 255-278, doi:10.1038/nprot.2016.169 (2017).

27 Kaschner, M. *et al.* Discovery of the first light-dependent protochlorophyllide oxidoreductase in anoxygenic phototrophic bacteria. *Molecular microbiology* **93**, 1066-1078, doi:10.1111/mmi.12719 (2014).

28 Heyes, D. J. *et al.* The first catalytic step of the light-driven enzyme protochlorophyllide oxidoreductase proceeds via a charge transfer complex. *The Journal of biological chemistry* **281**, 26847-26853, doi:10.1074/jbc.M602943200 (2006).

29 Sytina, O. A., van Stokkum, I. H., Heyes, D. J., Hunter, C. N. & Groot, M. L. Spectroscopic characterization of the first ultrafast catalytic intermediate in protochlorophyllide oxidoreductase. *Physical chemistry chemical physics : PCCP* **14**, 616-625, doi:10.1039/c1cp21713e (2012).

30 Schuck, P. A model for sedimentation in inhomogeneous media. I. Dynamic density gradients from sedimenting co-solutes. *Biophysical chemistry* **108**, 187-200, doi:10.1016/j.bpc.2003.10.016 (2004).

31 Kahn, A., Boardman, N. K. & Thorne, S. W. Energy transfer between protochlorophyllide molecules: evidence for multiple chromophores in the photoactive protochlorophyllide-protein complex vivo and in vitro. *Journal of molecular biology* **48**, 85-101 (1970).

32 Reinbothe, S., Pollmann, S. & Reinbothe, C. In situ conversion of protochlorophyllide b to protochlorophyllide a in barley. Evidence for a novel role of 7-formyl reductase in the prolamellar body of etioplasts. *The Journal of biological chemistry* **278**, 800-806, doi:10.1074/jbc.M209737200 (2003).

33 Heyes, D. J. & Hunter, C. N. Identification and characterization of the product release steps within the catalytic cycle of protochlorophyllide oxidoreductase. *Biochemistry* **43**, 8265-8271, doi:10.1021/bi049576h (2004).

34 Birve, S. J., Selstam, E. & Johansson, L. B. Secondary structure of NADPH: protochlorophyllide oxidoreductase examined by circular dichroism and prediction methods. *Biochem J* **317 (Pt 2)**, 549-555 (1996).

35 Zhang, Y. I-TASSER server for protein 3D structure prediction. *BMC bioinformatics* **9**, 40, doi:10.1186/1471-2105-9-40 (2008).

36 Canutescu, A. A. & Dunbrack, R. L., Jr. Cyclic coordinate descent: A robotics algorithm for protein loop closure. *Protein Sci* **12**, 963-972, doi:10.1110/ps.0242703 (2003).

37 Comeau, S. R., Gatchell, D. W., Vajda, S. & Camacho, C. J. ClusPro: an automated docking and discrimination method for the prediction of protein complexes. *Bioinformatics* **20**, 45-50 (2004).

38 Comeau, S. R., Gatchell, D. W., Vajda, S. & Camacho, C. J. ClusPro: a fully automated algorithm for protein-protein docking. *Nucleic acids research* **32**, W96-99, doi:10.1093/nar/gkh354 (2004).

39 Kozakov, D. *et al.* How good is automated protein docking? *Proteins-Structure Function and Bioinformatics* **81**, 2159-2166, doi:10.1002/prot.24403 (2013).
40 Kozakov, D., Brenke, R., Comeau, S. R. & Vajda, S. PIPER: an FFT-based protein docking program with pairwise potentials. *Proteins* **65**, 392-406, doi:10.1002/prot.21117 (2006).
41 Gabruk, M. *et al.* Insight into the oligomeric structure of PORA from A. thaliana. *Bba-Proteins Proteom* **1864**, 1757-1764, doi:10.1016/j.bbapap.2016.09.015 (2016).
42 Merkley, E. D. *et al.* Distance restraints from crosslinking mass spectrometry: Mining a molecular dynamics simulation database to evaluate lysine-lysine distances. *Protein Sci* **23**, 747-759, doi:10.1002/pro.2458 (2014).
43 Jacak, R., Leaver-Fay, A. & Kuhlman, B. Computational protein design with explicit consideration of surface hydrophobic patches. *Proteins* **80**, 825-838, doi:10.1002/prot.23241 (2012).
