# Supplementary material for: Consensus model of a cyanobacterial light-dependent protochlorophyllide oxidoreductase in its pigment-free apo-form and photoactive ternary complex
Source: Commun Biol. 2019 Sep 25;2:351. doi: 10.1038/s42003-019-0590-4 (PMC6761149; doi:10.1038/s42003-019-0590-4)
Supplement: Supplementary file 2 — Description of Additional Supplementary Files [file 42003_2019_590_MOESM2_ESM.docx]

**Supplementary Data 1:** Source data for main manuscript figures.

**Supplementary Data 2:** Source data for supplementary figures.

**Supplementary Movie 1:** Propagation of the sedimentation boundary of the *Te*LPOR holoprotein (1 mg ml^-1^) detected with a Nanolytics Instruments MWA ultracentrifuge.

**Supplementary Movie 2:** Propagation of the sedimentation boundary of the *Te*LPOR apoprotein (0.5 mg ml^-1^) detected with a Nanolytics Instruments MWA ultracentrifuge.
